# Expressivity of the key genes associated with seed and pod development is highly regulated via lncRNAs and miRNAs in Pigeonpea

**DOI:** 10.1038/s41598-019-54340-6

**Published:** 2019-12-03

**Authors:** Antara Das, Deepti Nigam, Alim Junaid, Kishor U. Tribhuvan, Kuldeep Kumar, Kumar Durgesh, N. K. Singh, Kishor Gaikwad

**Affiliations:** 1 ICAR- National Research Centre on Plant Biotechnology, New Delhi, India; 2 Division of Genetics, ICAR-IARI, New Delhi, India

**Keywords:** Plant development, Plant molecular biology, Seed development

## Abstract

Non-coding RNA’s like miRNA, lncRNA, have gained immense importance as a significant regulatory factor in different physiological and developmental processes in plants. In an effort to understand the molecular role of these regulatory agents, in the present study, 3019 lncRNAs and 227 miRNAs were identified from different seed and pod developmental stages in Pigeonpea, a major grain legume of Southeast Asia and Africa. Target analysis revealed that 3768 mRNAs, including 83 TFs were targeted by lncRNAs; whereas 3060 mRNA, including 154 TFs, were targeted by miRNAs. The targeted transcription factors majorly belong to WRKY, MYB, bHLH, etc. families; whereas the targeted genes were associated with the embryo, seed, and flower development. Total 302 lncRNAs interact with miRNAs and formed endogenous target mimics (eTMs) which leads to sequestering of the miRNAs present in the cell. Expression analysis showed that notably, Cc_lncRNA-2830 expression is up-regulated and sequestrates miR160h in pod leading to higher expression of the miR160h target gene, Auxin responsive factor-18. A similar pattern was observed for SPIKE, Auxin signaling F-box-2, Bidirectional sugar transporter, and Starch synthetase-2 eTMs. All the identified target mRNAs code for transcription factor and genes are involved in the processes like cell division, plant growth and development, starch synthesis, sugar transportation and accumulation of storage proteins which are essential for seed and pod development. On a combinatorial basis, our study provides a lncRNA and miRNA based regulatory insight into the genes governing seed and pod development in Pigeonpea.

## Introduction

Transcriptome sequences of an organism contains significant amount of non-coding RNAs (ncRNA) as compared to the protein-coding RNAs^1^. The housekeeping ncRNA such as rRNAs, tRNAs, small nucleolar RNAs, and small nuclear RNAs are expressed in tissues as well as stage independent manner; whereas regulatory ncRNAs which include microRNAs (miRNAs), small interfering RNAs (siRNAs) and long non-coding RNAs (lncRNAs) are expressed in stage and tissue-specific manner both^2^. Even though ncRNAs are not translated into proteins, they play a vital role in regulating the expression of other genes. The ncRNAs seems to regulate the expression at transcriptional, post-transcriptional as well as at chromatin level, thus participating in a complex regulatory network including the methylation controlled pathways. The miRNAs and siRNAs regulate gene expression by several mechanisms like suppression of gene transcription, degrading the target mRNA or inhibiting the translation of the target mRNA, whereas lncRNAs regulate gene expression in a different way, either by translation regulation, chromatin modification or via sequestering miRNAs in cells. The lncRNAs are transcribed by RNA polymerase II and III and are ≥200 nucleotides in length, having *cis*-acting as well as *trans*-acting sites and may also harbor both a 5′ cap and a poly-A tail in both plants and animal genomes. Based on genome localization, lncRNAs are classified into four types viz i) long intronic ncRNAs ii) long intergenic ncRNAs (lincRNAs) iii) Natural antisense transcripts (lncNATs) and iv) promoter lncRNAs^3^. Genome-wide discovery of lncRNAs was reported in many plant species such as *Arabidopsis*^4^, Maize^5^, Populus^6^, Rice^7^, Cucumber^8^, Medicago^9^, Wheat^10^, etc. The comparative analysis of lncRNAs showed limited conservation among the plants and animal species^4^. Transcriptome data are best suited for identification of tissue/stage-specific lncRNAs, where ≥200 nt length RNAs are selected, and protein-coding sequences are filtered out using blast search, and the remaining sequences with lack of protein-coding potential are considered as lncRNAs^6^. In *Arabidopsis thaliana*, a total of 6480 lncRNAs were reported using genome-wide screening of 200 transcriptome data^11^. The reported studies in strawberry, cotton, and chickpea reveals the regulatory role of lncRNAs on gene expression during fruit, fiber, and flower development respectively^12–14^. The lncRNAs COOLAIR and COLDAIR were required for epigenetic silencing of Flowering Locus C (FLC) during vernalization in *Arabidopsis thaliana*^15^. Rice lncRNA LDMAR has been reported to be involved in the regulation of photoperiod sensitive male sterility^16^. The lncRNAs Enod-40 and Mt-4 are reported to be involved in nodulation of *Medicago truncatula*^17^. Along with the regulation of primary plant metabolism, lncRNAs also play a crucial role during biotic and abiotic stresses^9,18,19^.

Micro RNAs (miRNAs) are a class of ncRNAs 20–24 nt in length and transcribed by RNA polymerase II. It is a well-characterized class of ncRNA that regulate many biological processes including signal transduction, growth, development, hormone signaling, homeostasis, innate immunity, and response to different abiotic and biotic stresses in plants^20^. They are primarily involved in gene regulation by either degradation or inhibition of translation of target mRNA. Small RNA sequencing is one of the best methods for identification of miRNAs present in the tissue/organism at a particular stage of growth^20,21^. Alternatively, many studies have utilized ESTs, CDS, genomic, and transcriptome data to dig out miRNAs. The use of these data for mining of miRNAs provide an opportunity for effective utilization of available data present in the database but may yield incomplete information about expressed miRNAs at a particular stage/tissue of an organism^22^. The non-redundant dataset like EST, c-DNA and GSS sequences of *A*. *thaliana* and *P*. *vulgaris* were also used for identification of the miRNAs^23,24^. Studies carried out in various crop species like cereals, legumes, tuber, fruit, bio-fuel, beverage, etc. showed that miRNAs are conserved across the plant genera^20–25^. In rice, the miR393 has been reported to target auxin receptor gene (TIR1 and AFB2) which are related to drought response, high tillering and early flowering^26^. The plant growth and development related genes for squamosa binding protein and ARF transcription factors were targeted by miR156 and miR160, respectively in maize^27^. Likewise, maize genes for ARF and NF-YA transcription factors were targeted by miR167 and miR169, respectively. ARF and NF-YA transcription factor are known to be involved in growth and development as well as many stress-responsive processes^26,27^. It was reported that in *A*. *thaliana*, mutant for miRNA biogenesis, processing, and loading showed embryo lethality, severe embryo defects, and abnormal seedling formation after germination. The timing and stage of individual miRNAs expression are also important for proper seed and pod development of plants^20^.

The lncRNAs act variably due to its long length, forming a super-secondary structure which provides a large number of binding sites for DNA, RNA, Protein, TFs and are also able to recruit different types of regulatory elements. The lncRNAs regulate the gene expression at either transcriptional or post-transcriptional level. For the transcriptional level of regulation, lncRNAs act as a mediator to recruit chromatin modifiers, transcription factors, activator proteins and/or repressor protein to the chromatin for its modification, resulting in activation or suppression of target gene expression^28^. The post-transcriptional gene regulation through lncRNAs is effected out through the mechanism of endogenous target mimic (eTM)^29^. The eTMs are the interaction between lncRNAs and miRNAs, and this interaction regulates the expression of miRNA targeted genes. The lncRNAs have miRNA binding sites; the presence of miRNA specific lncRNA in the cell sequesters the miRNAs; leading to higher expression of miRNA targeted genes. On the other hand, in the absence of lncRNAs, the number of miRNAs get elevated, leading to suppressed expression of their target gene^29,30^. The mechanism behind eTM mediated gene regulation was well characterized in *Arabidopsis thaliana* using gene PHO2 and lncRNA IPS1. Both PHO2 and IPS1 showed elevated gene expression during phosphate starvation condition. It is well known that PHO2 is involved in inorganic phosphate (Pi) homeostasis and regulates through miR399 at a post-transcriptional level^29^. It was found that lncRNA IPS1 and mRNA of PHO2 has 23 nucleotide common binding site for miR399. In the presence of IPS1, all the molecules of miR399 showed a strong affinity to bind with IPS1, leading to up-regulation of PHO2 gene. In the absence of IPS1, miR399 is free to suppress the expression of PHO2^29^. The role of lncRNAs in seed and pod development has not been studied yet, but its role in flowering is well characterized in chickpea and strawberry^12,14^.

*C*. *cajan* seeds are an affluent (20–23%) resource of protein which forms a perfect blend in combination with cereals for providing a balanced human diet, particularly in South East Asia. Seed development is a genetically complex as well as the metabolically dynamic event in the plant life cycle. In the past, various studies has been reported which have shown that hormonal and metabolic regulation play role in seed development stages of *C*. *cajan*^31,32^. The accessibility of *C*. *cajan* draft genome and transcriptome sequences offers the scope to carry out advanced genomic research. Recently, genome-wide identification of lncRNAs and miRNAs in *C*. *cajan* was reported by Nithin *et al*.^33^, but insight into their regulatory function in seed and pod development in *C*. *cajan* is not clear.

In the present study, a comprehensive identification and characterization of lncRNAs and miRNAs were carried out using seed and pod specific transcriptome data of *C*. *cajan*, to understand their probable regulatory role during seed and pod development.

## Result and Discussion

### Identification of lncRNAs in *C*. *cajan* from seed and pod developmental stages specific transcriptome data

**Figure 1:**
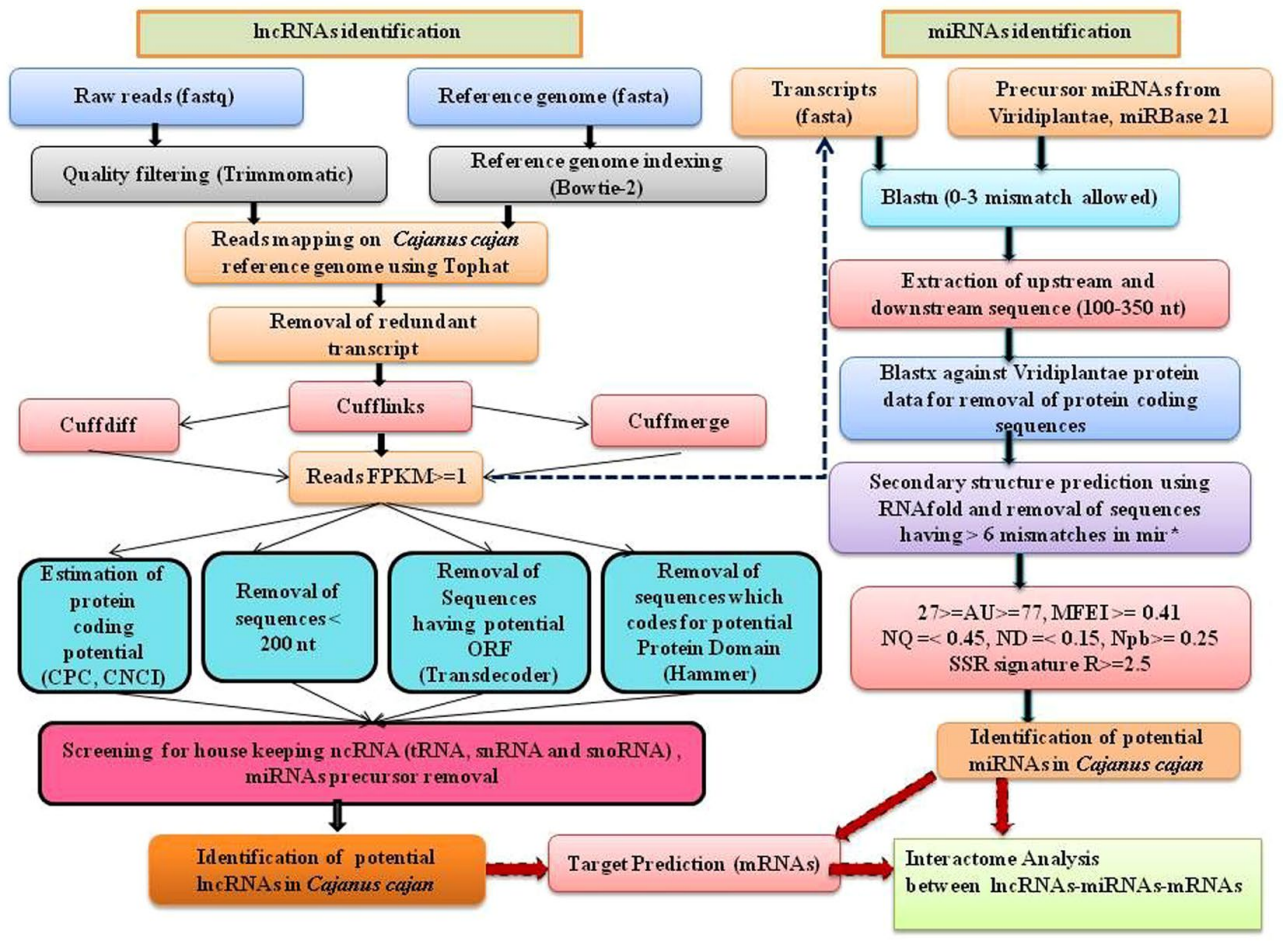
The pipeline used for identification of lncRNAs and miRNAs in *Cajanus cajan*.

To identify lncRNA and miRNAs related to seed and pod development in *C*. *cajan*, we used the available transcriptome data from NCBI (https://www.ncbi.nlm.nih.gov/), (Supplementary S1). A total of 1372 million raw reads were obtained from leaf and seed/pod tissues at 0 DAA (days after anthesis), 10 DAS (seed tissue-days after anthesis), 20 DAS, 30 DAS, 10 DAP (pod tissue- days after anthesis), 20 DAP and 30 DAP of Asha cultivar of *C*. *cajan*. After trimming, 1336 million clean reads were mapped on the *C*. *cajan* reference genome using TopHat2 and Bowtie programme^34^. A total of 1254 million (91.41%) reads were finally mapped on the reference genome. The FPKM of each gene and its corresponding isoforms was calculated using Cufflinks program^35^. A total of 100446 transcripts with 63295 loci were obtained after Cuffmerge with an average length of the transcripts at 3849 bp. All the obtained transcripts were filtered based on its length and exon numbers and 41971 transcripts with length ≥200 bp were selected for further processing. The coding potential of all the selected transcripts was checked through CNCI and CPC; finally, 14533 transcripts with the score ≤−0.5 were selected. After homology search, using Transdecoder, Hammer, and blastx with complete protein dataset from Swissport in Pfam, only 3313 transcripts remained. Finally, rRNA and all other groups of ncRNAs were also removed, and 3019 transcripts were identified as potential lncRNAs. The pipeline used for identification of lncRNAs and miRNAs in *C*. *cajan* is shown in Fig. 1.

### Characteristic features of predicted lncRNAs

**Figure 2:**
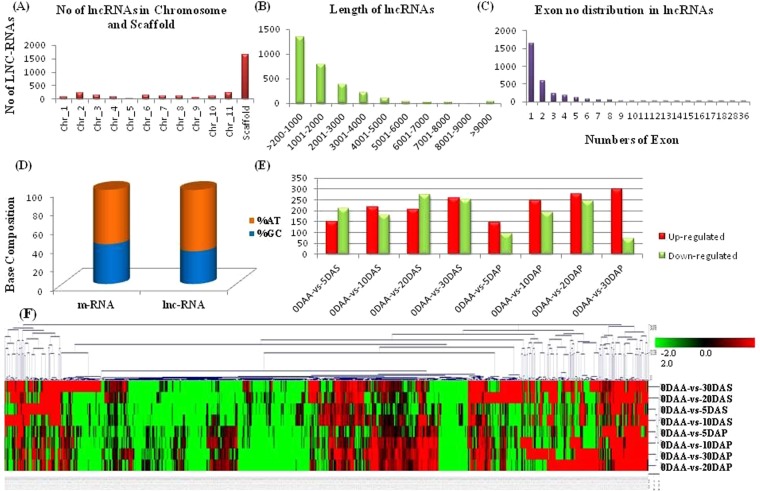
Physical properties of Predicted lncRNAs in *C*. *cajan* (**A**) Chromosome and scaffold wise distribution of lncRNAs. (**B**) Length distribution of lncRNAs. (**C**) Exon number distribution in lncRNAs. (**D**) Comparison of AT and GC % among lncRNAs and mRNAs. (**E**) Number of differentially expressed (DE) lncRNAs in the different combination of tissues. (**F**) Heat map for DE lncRNAs in different seed and pod tissues as compare to 0 DAA (log2 fold change).

The average length of *C*. *cajan* lncRNAs were 1784nt, whereas the maximum number of lncRNAs were in the range of 200–1000 nt. A total of 1638 (54%) of lncRNAs obtained in *C*. *cajan* were mono-exonic, followed by 580 (19%) di-exonic and 232 (7.7%) were tri-exonic. A continuous variation of exon numbers from 1–18 was observed except for two lncRNA having 28 and 36 exons. It was noted that lncRNAs are AU-rich as compared to mRNAs. Similar kind of observations has been reported in *Arabidopsis*, Maize, Populus, and Rice^4–7^. The chromosome-wise distribution of lncRNAs in *C*. *cajan* genome shows that chromosome 11 has the highest number of lncRNAs, while chromosome 5 has lowest. The characteristic features of *C*. *cajan* lncRNA are provided in Supplementary S2 and Fig. 2.

### Identification of differentially expressed lncRNAs between 0 days after pollination (DAP) bud vs. all other developmental stages of seed and pod development

A total of 1159 differentially expressed (DE) lncRNAs were identified among all samples. DE [FC value > 2 and <−2 (p-value less than 0.005 and q-value less than 0.01)] lncRNAs were observed in tissue-specific manner. Highest DE lncRNAs were observed in 0 DAP vs. 30 DAP (576) followed by 0 DAP vs. 20 DAP (523) and 0 DAP vs. 30 DAS (511) with many DE lncRNAs common between them. The detailed expression pattern of differential expression between the tissues is shown in the heatmap. The lncRNAs expression in each tissue varied from 17.03 fold to −16.67 fold (Fig. 2; Supplementary S3).

### Identification of lncRNAs targeted mRNAs including TFs

The lncRNAs control gene expression in two ways; viz. they positively regulate the target gene by sequestering gene-specific miRNA or via accommodating other regulatory elements in 5′UTRs of the genes. The super-secondary structure of lncRNAs provides the binding sites for miRNA, mRNA, TFs, and other regulatory elements. Hence, the formation of the super-secondary structure of lncRNA helps to bring together all transcription factors, miRNA, and other regulatory elements to influence gene expression in a positive manner or vice versa. In *C*. *cajan* seed and pod tissues, a total of 1,660 lncRNAs were found to have potential binding sites for 3768 mRNAs (Dng less than equal to −50), and among these, 83 were TFs belonging to 20 families (Supplementary S4).

Pearson correlation coefficient was calculated for each pair of interacting lncRNA, and the target mRNA. The correlation coefficient was calculated between the expression changes of lncRNAs and their target genes in different developmental stages of seed and pod development viz. 5DAS, 10DAS, 20DAS, 30DAS, 5DAP, 10DAP, 20DAP and 30DAP (in terms of log2 fold changes). It was observed that among all the possible interaction with certain binding energy (cut-off value Dng less than equal to −50), 81% of the total lncRNA and its targets mRNA showed a correlation. Among the interacting pair, 79% have shown a positive correlation and 21% negative correlation. Overall 51% pair belongs to the correlation range of +0.8 to +1 and −0.8 to −1, 63% pair belongs to the correlation range of +0.6 to +1 and −0.6 to −1, and 69% pair belongs to the correlation range of +0.5 to +1 and −0.5 to −1(Supplementary S4).

The identified transcription families were MYB, Ethylene responsive TFs, bHLH, Nuclear TF-Y, TFIID, GATA, WRKY, etc. which are reported to be actively involved in embryo, and seed development activities of plant^36^. The identified lncRNAs target mRNAs are involved in the essential biological functions such as chromatin modification (topoisomerases), DNA replication (DNA Helicase, DNA ligase), transcription (transcription initiation, elongation factor etc.), cell division (expansion), vascular sorting, trafficking proteins, heat shock proteins, callose and cellulose synthase. Most of the mRNAs are related to hormone biosynthesis and signaling (auxin-induced protein, gibberellin-regulated proteins, auxin-responsive F-box proteins), serine/threonine-protein kinase, F-box-LRR repeat protein, and B-box zinc fingers. The lncRNAs also target the mRNAs for transporters (ABC transporters, sodium/potassium transporters, and sugar transporters). Many other mRNAs coding for enzymes like glycosyltransferase, glycerol-3-phosphate 2-O-acetyltransferase GDSL esterase/lipase were also a target for lncRNAs. The function of lncRNA targeted mRNAs are diverse and are required to carry out the routine as well as a specialized role such as seed development, maturation, and storage of reserved food in the seed^36^.

### Real time-PCR validation of correlated lncRNAs and mRNAs pairs

**Figure 3:**
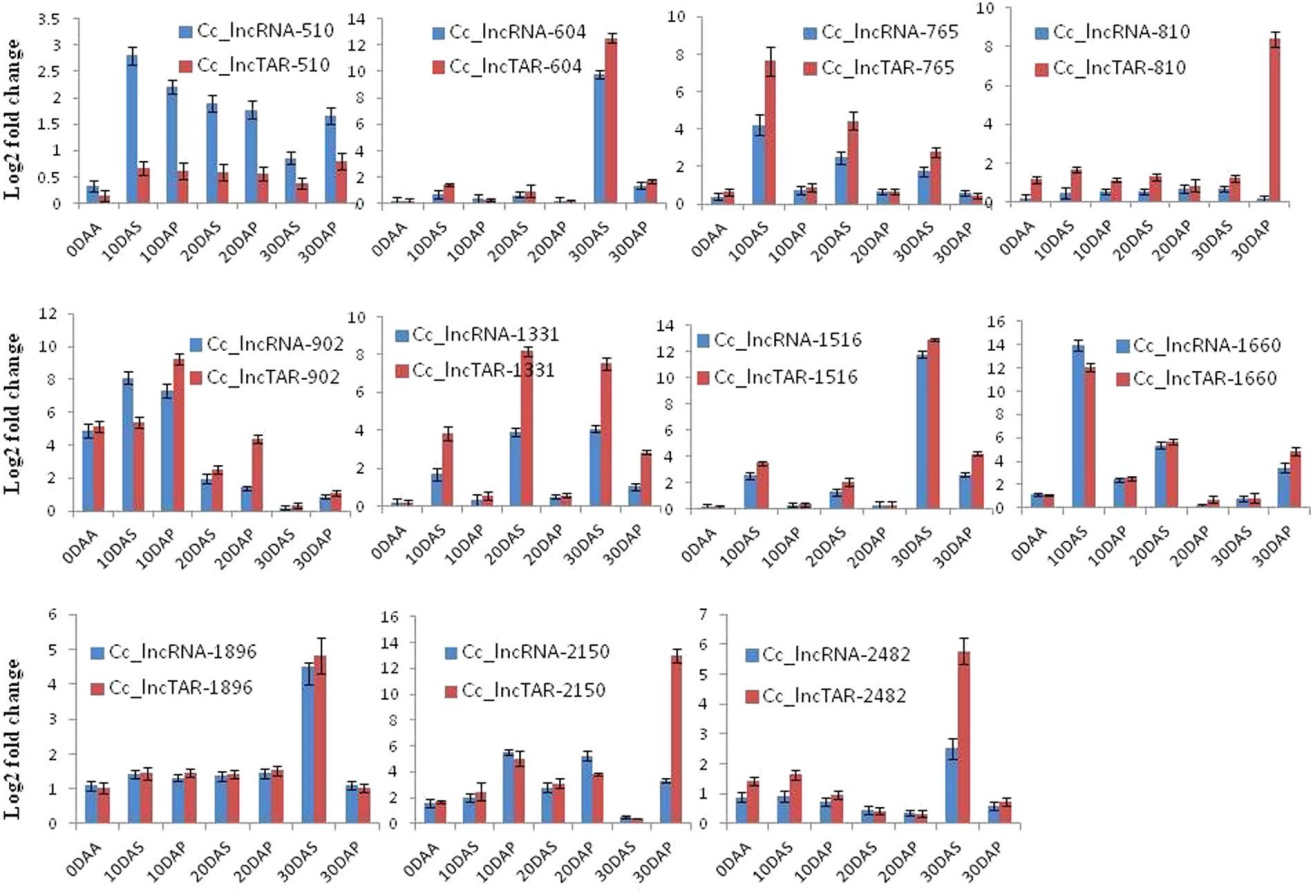
The quantitative RT-PCR analysis of lncRNAs and their target mRNAs involved in seed and pod development; blue color represent lncRNA, and the red color represents their target mRNAs.

To explore expression behavior of tissue and stage specificity, we performed expression analysis of lncRNAs along with its target genes in 7 different tissues (0 DAA, 10 DAS, 20 DAS, 30 DAS, 10 DAP, 20 DAP, and 30 DAP) using real-time PCR (Fig. 3).

The results of real-time PCR analysis revealed that the Cc_lncRNA-510 and its target mRNA Cc_lncTAR-510, a VQ motif-containing protein showed the highest expression after 10 DAA in both seed and pod tissues, and it was sustained up to seed and pod maturity. The VQ family proteins were known to be involved in seed development, photomorphogenesis and biotic, and abiotic stresses in plants^37^. VQ motif-containing proteins were seen to interact with WRKY transcription factor and showed a potential role in transcriptional regulation of genes involved in fruit ripening^38^. The Cc_lncRNA-604 and its target mRNA Cc_lncRNAtar-604, a Late Embryogenesis Abundant (LEA) protein showed the highest expression during 30DAS. It is the member of LEA protein which is known to be expressed during embryo maturation and seed development. During the seed maturation, LEA protein has a significant role in mitigating water loss and maintaining cellular stability within the desiccated seed^39^. The Cc_lncRNA-765 and its target mRNA Cc_lncTAR-765, a carboxy peptidase-like mRNA expression was observed to be consistently higher in seed tissue as compared to the pod tissue. Higher expression was seen in 10 DAS, which gradually reduced during the later stages of seed and pod development. In seed and pod development stages, both Cc_lncRNA-765 and its target mRNA expression level are highest at 30 DAP. Serine carboxypeptidase (SCP) is one of the major groups of enzymes involved in functional protein maturation through catalyzing proteolysis. Previous studies in Arabidopsis and rice showed that SCP expresses in roots, leaves, flowers, and predominately in seed and siliques^40^. Cc_lncRNA-810 and its target Cc_lncTAR-810, an F-box/Kelch repeat-containing protein showed consistent expression throughout the seed and pod development with the highest expression of Cc_lncTAR-810 and minimal expression of Cc_lncRNA-810 at 30DAP. The expression pattern of lncRNA and it’s target showed negative correlation, which suggest that Cc_lncRNA-810 negatively regulate the expression of its target mRNA. F-box/Kelch repeat-containing protein was involved in the regulation of light, flower initiation, and grain filling in rice^41^. The Cc_lncRNA-902 and its target Cc_lncTAR-902, which codes for Aquaporin TIP4 showed the highest expression 10 DAP and 10 DAS with consistent lower expression at later stages. The members of aquaporins family proteins are engaged in the transport of water and small neutral solutes across the cell membrane. It was also reported that some of the members of the aquaporin family-like TIP3, TIP2, etc. showed upregulated expression in the developing seed and has a possible role in the transportation of organic compound during seed maturation^42^. The Cc_lncRNA-1331 and its target Cc_lncTAR-1331, Loricrin-like mRNAs showed consistent expression throughout the seed and pod developmental stages with a peak of expression at 20DAS. Both lncRNAs and its target showed higher expression in seed tissues as compared to pod tissues. Loricrin is involved in cornified cell envelope development in animal^43^, whereas its function is not elucidated in plants. The Cc_lncRNA-1516 and its target Cc_lncTAR-1516, codes for P24 oleosin like protein and showed maximum expression at 30 DAS. Oleosin is an integral component of oil bodies and has a structural role in stabilizing the lipid body during desiccation of the seed^44^. The Cc_lncRNA-1660 and its target Cc_lncTAR-1660, which codes for hydrolase enzyme showed higher expression at 10DAS and 20DAS and declined expression at 30DAS. It was observed that the appearance of hydrolase during milk gain stage was at the peak and decreased at seed maturation stage^45^. Likewise, Cc_lncRNA-1896 and its target mRNA CclncTAR-1896, a beta-conglycinin, the beta chain-like peptide expression pattern was positively correlated (0.99) and found to be at the highest expression level at 30 DAS. The reported studies suggest that beta-conglycinin and glycinin are the major proteins found in soybean seed development^46^. The Cc_lncRNA-2150 and its target Cc_lncTAR-2150, which codes for dirigent protein was highly expressed in pod tissues as compared to seed and showed a peak of expression at 30DAP. It is involved in deposition of defense cyanogenic glucosides in plants. In the flax seeds, synthesis of dirigent protein occurred during seed development, which was further utilized for lignin and cyanogenic glucoside formation^47^. Cc_lncRNA-2482 and its target mRNA Cc_lncTAR-2482, a *Glycine max* maturation associated protein (MAT-9) expression was highest in 30 DAS sample. It is either present at low levels or absent in leaves, but its level gradually increases in seed tissues as the seed enters maturity. The MAT-9, a maturation-associated plant-specific protein also known as DHN1 (Type 2 LEA protein) is commonly associated with seed maturation, seed dehydration, and desiccation tolerance during seed maturation^48^.

### Comparative GO analysis of lncRNAs and its targeted mRNAs

**Figure 4:**
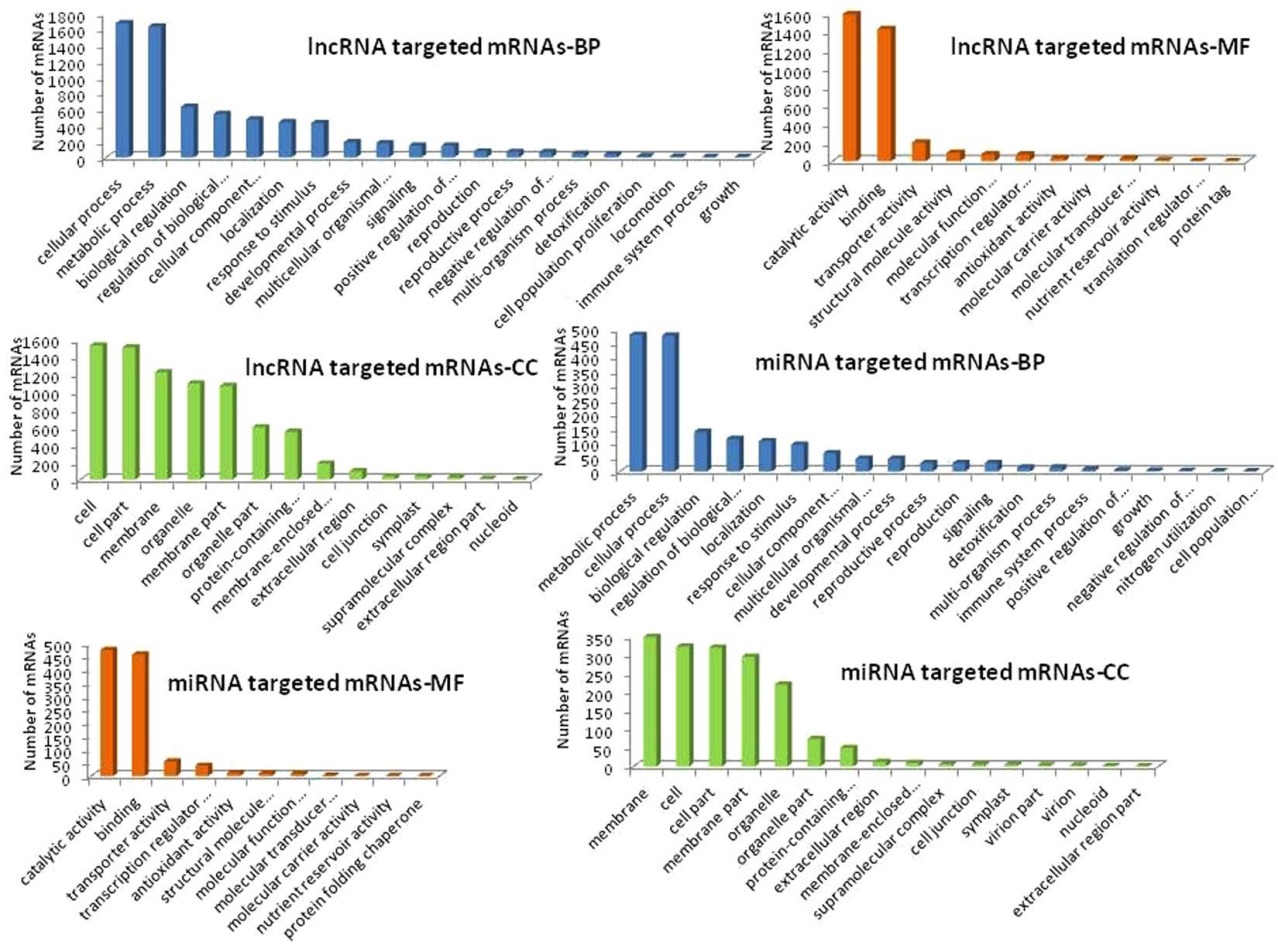
Gene Ontology (GO) analysis of lncRNAs targeted mRNAs and miRNAs targeted mRNAs; BP- Biological Process, MF- Molecular Function, CC- Cellular Component.

In GO (gene ontology) study at the level of biological process, comparative analysis between lncRNAs (Supplementary Fig. 1, Supplementary S5), and lncRNA’s target (Fig. 4, Supplementary S6), revealed that 49% and 48.40% of total lncRNAs and lncRNA’s targets respectively were involved in a metabolic and cellular process. It was observed that 30% of lncRNAs and 30.57% of lncRNA’s targets were associated with biological regulation, cellular component organization, biogenesis, and localization. Rest 21.7% of lncRNAs, and 21.1% of lncRNA’s targets are involved in the biological process like localization, response to a stimulus, signaling, developmental process, reproductive process, growth, and other cellular processes.

The comparative analysis at the level of the molecular function of lncRNAs (Supplementary Fig. 1, Supplementary S5), and lncRNA targeted mRNAs (Fig. 4, Supplementary S6), showed that majority of them are involved in a catalytic activity which covers 43.55% and 44.46% in lncRNAs and its targets respectively. Binding covers 38.8% of lncRNA and 40.02% of lncRNA’s targets. Rest 18% of lncRNAs, and 15.53% of lncRNA’s targets were involved in activities like the transporter, molecular function regulator, signal transduction, and protein binding. The comparative analysis at the level of localization of lncRNAs (Supplementary Fig. 1, Supplementary S5), and lncRNA targeted mRNAs (Fig. 4, Supplementary S6) also showed a positive correlation. In the cell and cell part, 37.22% and 38.15% of lncRNAs and lncRNAs target mRNA were residing respectively while in membrane and membrane parts 36.1% and 28.79% of lncRNAs and lncRNAs target mRNA were present, respectively. The comparative analysis at three different levels suggests that lncRNAs and its targets coexist in the cell because their functionalities are interdependent and linked.

### Identification and characterization of miRNAs involved in seed and pod development

**Figure 5:**
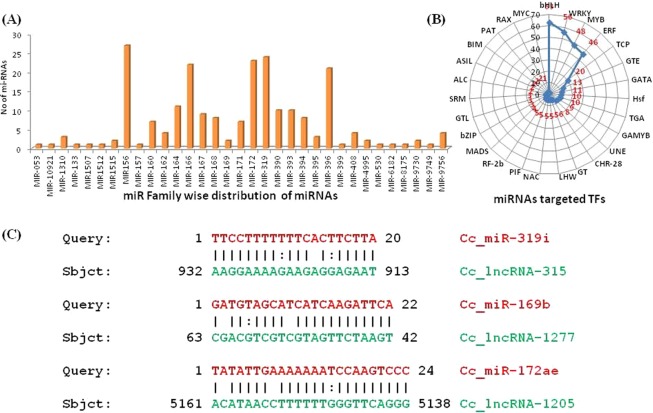
(**A**) miR Family wise distribution of miRNAs (**B**) Statistics of miRNAs targeted TFs families. (**C**) eTM binding sites for lncRNAs and miRNAs.

A total of 227 miRNAs were identified using transcriptome data obtained from various stages of seed and pod development in *C*. *cajan* using the pipeline depicted in Fig. 1. The miRNA identification pipeline used in the study has been computationally validated with high specificity and sensitivity in *A*. *thaliana*^23^, *P*. *vulgaris*^24^, and *C*. *cajan*^33^. All physical properties of identified miRNAs are provided in Supplementary S7. All the identified miRNAs are novel and represent 33 different families, the maximum numbers of miRNA fall under miR156 family (27) followed by miR169 (24), miR172 (23), and miR166 (22). A similar type of family-wise distribution of miRNA was reported in *P*. *vulgaris*^24^. The length of predicted mature miRNAs ranges from 14 to 25 nt. It was observed that 29 signature SSRs were present in these miRNA families, among them UUU signature frequency was highest followed by AAA. The complete information about the miR-family wise distribution of predicted miRNAs is shown in Fig. 5(A) and Supplementary S8.

### Identification of mRNA targeted by miRNA

All the identified 227 miRNA were subjected to psRNATarget software for prediction of their target mRNAs. The 5′UTR and 3′UTR region of mRNA sequence from *C*. *cajan* database were used as miRNAs targets. We chose the UTR region of genes for target prediction analysis with the assumption that the miRNAs usually act on the UTR regions of the genes^20^. Total, 3060 potential mRNAs targets were identified and further classification of all target mRNA showed that 154 mRNA were coding for TFs, representing 21 TFs families including bHLH, WRKY, MYB, ERF, etc. (Fig. 5(B) and Supplementary S9). Studies on *Arabidopsis* mutants for carpel margin tissues and fruit patterning showed that bHLH families of transcription factors are fundamentally crucial for developmental and environmental responses^49^. The MYB family TFs (MYB-118) is actively involved in *Arabidopsis* endosperm development and seed maturation^50^. In our analysis, we found that MYB (14.5%) family and bHLH (12.5%) family mRNAs are targeted by Cc-miRNAs suggesting that Cc-miRNA may be involved in regulating seed and pod development activity by modulating the expression of these two TF family members.

**Figure 6:**
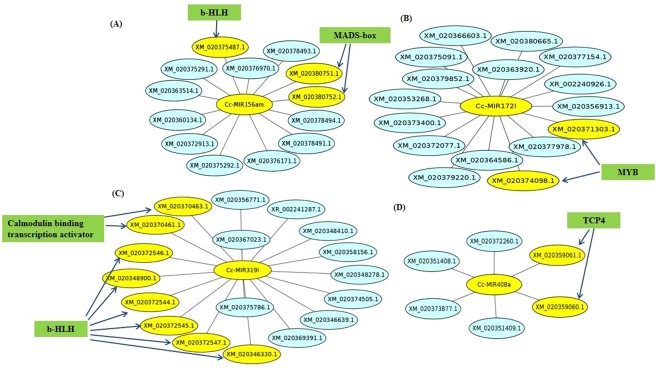
miRNA targeted mRNAs including TFs, centre of the interaction is miRNA and in periphery blue circles represent the mRNAs and yellow circles represent the TFs, (**A**) miR156am-bHLH and miR156am-MADS-box TFs interaction (**B**) miR172l-MYB TF interaction (**C**) miR319l-bHLH TF and miR319l-Calmodulin-binding transcription activator interaction (**D**) miR408a-TCP4 TF interaction.

The results of Cytoscape (Fig. 6) showed the interaction pattern between miRNAs and their targets, and it was observed that one miRNA could interact with many mRNAs. The miR156 showed its interaction with bHLH and MADS-box TF along with other mRNAs. miR-172l interacts with MYB TF, and miR408a interacts with TCP4 TF. MiR319l can target Calmodulin-binding transcription activator and bHLH TF. It is well known that relative level of miR156, miR172 helps in juvenile to reproductive phase transition^51^. MYB, MADS-box, and bHLH are involved in growth and development during different seed stages. All the miRNAs targets (3′ UTRs and 5′ UTRs) with their interacting mode is given in Supplementary S9. All the predicted miRNAs and their target mRNA interacting pattern is visualized via Cytoscape and shown in Supplementary Fig. S2.

### GO annotation of miRNAs targeted mRNAs

We performed Blast2Go analysis to annotate the predicted mRNAs, which are targeted by miRNAs. The molecular function, biological processes, and cellular components of the targets are shown in Fig. 4 (Supplementary S10). Under the Biological process, the majority of the targets viz. 56.08% were involved in the metabolic and cellular process. Around 41.05% were involved in biological regulation, localization, regulation of the biological process, response to a stimulus, signaling, cellular component organization or biogenesis, multicellular organismal process, developmental process, reproduction, reproductive process, and growth. Rest 2.86% was associated with detoxification, positive and negative regulation of the biological process, cell population proliferation, immune system process, and nitrogen utilization.

Under molecular functions, catalytic activity covered 44.59%, binding covered 43.01% of transcripts and rest of 12.38% were involved in transporter activity, transcription regulator activity, structural molecule activity, antioxidant activity, molecular function regulation, molecular transducer activity, molecular carrier activity, nutrient reservoir activity and protein folding chaperone.

The targets localized in different cellular components, among them a large number of proteins localize in cell and cell part (38.25%), membrane and membrane parts (38.38%), organelle and organelle part (17.54%) and rest 5.12% in protein-containing complex, extracellular region, membrane-enclosed lumen, supramolecular complex, cell junction, microtubules, symplast, virion and nucleoid.

### Identification of lncRNAs as candidate endogenous target mimics

Endogenous target mimics (eTMs) is build up through pairing among miRNAs and lncRNAs occurring in cells^29,30^. The psRNATarget analysis revealed that a total of 227 miRNAs target 3060 mRNAs, among these 227 Cc-miRNAs, 139 Cc-miRNAs were also act as target for 703 Cc_lncRNAs, suggesting that miRNA could be involved in interacting with both lncRNA and miRNA. The results obtained in our study supports the possibility that the formation of endogenous target mimics (eTMs) between Cc_lncRNAs and miRNAs regulates the gene expression profile during seed and pod development.

The result of endogenous target mimics (eTMs) analysis using an in-house script and psRobot software revealed that 302 lncRNAs interact with 114 miRNAs and formed eTMs (Fig. 5(C) and Supplementary S11). The obtained results support the hypothesis that lncRNA regulates the function of mRNAs indirectly by the mechanism of endogenous target mimic (eTMs) via miRNAs. Details of eTMs and their target mRNAs along with their function are provided in Table 1. The miRNA and lncRNA interacting network via eTMs is also executed and visualized via Cytoscape in Supplementary Fig. S3.

**Table 1:** eTMs with their target mRNA expressed during seed and pod development of *C*. *cajan*.

SN.	eTMs		eTMs target	The function of eTM target	References
	lncRNAs	miRNA			
1	Cc_lncRNA-2830	Cc-miR-160h	XM_020377020	*C*. *cajan* auxin response factor 18-like mRNAs	Li *et al*.^67^
2	Cc_lncRNA-1034	Cc-miR-164h	XM_020382865	*C*. *cajan* guanine nucleotide exchange factor SPIKE1	Zhang *et al*.^52^
3	Cc_lncRNA-2988	Cc-miR -164-i	XM_020371081	*C*. *cajan* protein Auxine Signaling F-BOX 2-like (LOC109808190)	Leyser.^53^
4	Cc_lncRNA-2472	Cc-miR -319i	XM_020376546	*C*. *cajan* bidirectional sugar transporter SWEET5-like (LO)	Chen.^54^
5	Cc_lncRNA-2928	Cc-miR -156as	XM_020364576	*C*. *cajan* UDP-glucose: glycoprotein glucosyl transferase (LOC109803127)	Blanco *et al*.^55^
6	Cc_lncRNA-703	Cc-miR -166z	XM_020347729	*C*. *cajan* late embryogenesis abundant protein like (LOC109788880) mRNA	Manfre *et al*.^39^
7	Cc_lncRNA-1921	Cc-miR -9756e	XM_020376117	*C*. *cajan* somatic embryogenesis recerptor kinase 2 (LOC109812199), mRNA	Hecht *et al*.^68^
8	Cc_lncRNA-1277	Cc-miR -156aq	XM_020384165	*C*. *cajan* sugar transport protein 10-like (LOC109818634)	Weber *et al*.^69^
9	Cc_lncRNA-2117	Cc-miR -166f	XM-020363260	*C*. *cajan* granule-bound starch synthase 2, chloroplastic/amyloplastic like (LOC109802053) mRNA	Patron *et al*.^70^
10	Cc_lncRNA-1819	Cc-MIR-1310c	XM_020351344	*C*. *cajan* NAD(P)H-quinone Oxidoreductase N subunit	Eugeni *et al*.^71^
11	Cc_lncRNA-1207	Cc-MIR-156ag	XM_020384198	*C*. *cajan* glycerol-3-phosphate2-O-acyltransferase 6-like	Vigeolas *et al*.^72^
12	Cc_lncRNA-2596	Cc-MIR-393m	XM_020382918	*C*. *cajan* Probable polygalacturonase (LOC109817608)	Ogawa *et al*.^73^

Fan *et al*.^30^, identified eTMs in degradome data of maize and reported that expression of lncRNAs disrupted the miRNA-mRNA regulation. They found 34 lncRNAs; having a binding site for 33 miRNAs involved in the regulation of various pathways like hormone signaling, sugar, fatty acid biosynthesis, sugar transportation, starch biosynthesis, etc^52–55^. It was observed that two or more Cc_lncRNAs have interacted with a single Cc-miRNA via eTMs. Cc_lncRNA-1819, Cc_lncRNA-2051 and Cc_lncRNA-2889 were endogenously targeted by Cc-miR1310c. Similarly, Cc_lncRNA-1277 and Cc_lncRNA-1740 are targeted by Cc-miR156ar; Cc_lncRNA-1741, Cc_lncRNA-2342 and Cc_lncRNA-2845 are targeted by Cc-miR167m; Cc_lncRNA-1854 and Cc_lncRNA-2988 are targeted by Cc-miR172aa; Cc_lncRNA-1657, Cc_lnc_1901 and Cc_lncRNA-2732 targeted by Cc-miR396w. Similar kind of interaction pattern of the eTMs has also been observed in *Cyamopsis tetragonoloba*^56^.

### Validation of lncRNAs, miRNAs and eTMs targets through expression analysis

**Figure 7:**
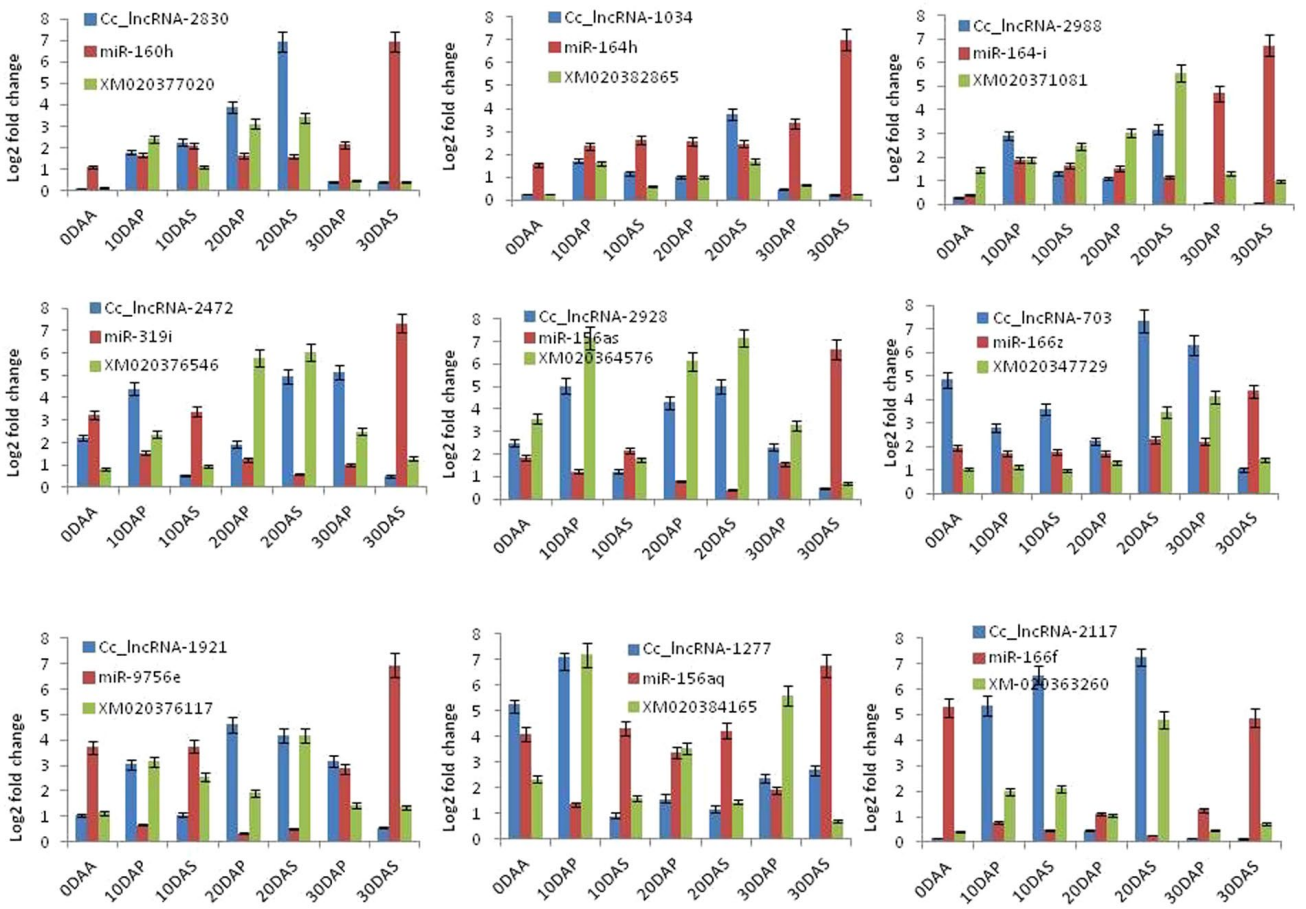
Real time-PCR analysis of miRNAs-lncRNAs as eTMs and miRNAs probable targets involved in seed and pod development; blue color represent lncRNA, red color represents miRNA, and green color represents mRNAs, targets of the corresponding miRNA.

The expression patterns of predicted eTMs were validated using real-time PCR by taking nine pairs of eTMs, which are linked to seed and pod development pathways (Fig. 7). The real-time expression analysis revealed that the eTM (Cc-miR160h–Cc_lncRNA-2830) regulate the mRNA level of XM_020377020, which codes for ‘Auxin response factor 18-like (ARF-18)’ protein. Cc-miR160-h expression was minimal at 0 DAA and was progressively up-regulated at the later stages of seed and pod development, and found to be highest at 30 DAS. The expression pattern of Cc-miR160h target mRNA XM_020377020 was minimal at 0 DAS and maximum at 10 DAS and 20 DAS and reduced by 30 DAS. The obtained result does not support the existing hypothesis that only Cc-miRNA160h regulates the expression of XM_020377020, because, at 10 DAS and 20 DAS, sufficient amount of Cc-miR160h were present in the cell. Cc_lncRNA-2830 showed minimal expression at 0 DAS and progressively increased its expression up to 20 DAS with a decline towards 30 DAS. In conclusion, it can be assumed that in the presence of high amounts of Cc-miR160h at the 10 DAS and 20 DAS, expression of XM_020377020 was found to be high, because at same time abundance of Cc_lncRNA-2830 sequesters Cc-miR160h and XM_020377020 transcript could be freely available for translation. At 30 DAS, Cc_lncRNA-2830 expression is on a decline, and Cc-miR160h expression is up-regulated as a result, which could be leading to degradation of XM_020377020. It was observed that seeds and pods reach its maximum size and shape about 20 days after anthesis by vigorous cell division and cell growth, in which the ARF-18 factor is needed because it helps in auxin-regulated cell growth and development^57^.

Similar type of interaction pattern was also observed in eTM (miR164h–Cc_lncRNA-1034) for XM_020382865, a guanine nucleotide exchange factor SPIKE1; (miR164i–Cc_lncRNA-2988) XM-020371081, a auxin signaling F-box 2; (miR319i–Cc_lncRNA-2472) XM-020376546, a Bi-directional sugar transporter; (miR156as–Cc_lncRNA-2928) XM-020364576, UDP-glucose-glycoprotein-glycosyltransferase; (miR1662–Cc_lncRNA-703) XM-020347729, a LEA protein; (miR9756e–Cc_lncRNA-1921) XM-020376117, a Sugar transporter; (miR156aq–Cc_lncRNA-1277) XM-020384165, Sugar transporter; and (miR166F–Cc_lncRNA-2177) XM-020363260, a Granule-bound Starch synthase-2.

Transcription factor SPIKE, also known as NAL1 in rice, regulates the development of seed/spike and is responsible for an increase in spike number and seed size^58^. In our study, it was observed that its expression was higher in 10DAP and 20DAS, which is the most critical stage of seed and pod development where most of the photosynthate produced by plant starts to accumulate in seed tissues as a sink. The auxin signaling F-box 2 is linked with positive regulation of auxin biosynthesis, which is involved in seed enlargement and seed size enhancement^53^. Bi-directional sugar transporters were involved in the transport of sugar molecule from one part of the plant to other^54^. Our study confirmed that after 10 and 20 days of anthesis in both seed and pod, bidirectional sugar transporter is activated to accumulate the plant photosynthetic sugar to seed as a sink. UDP-glucose-glycoprotein-glycosyltransferase is involved in glycosylation of protein moieties. Higher expression of this protein indicated that it gets collected in the seed as sink and gets glycosylated^59^. In case of LEA (Late embryogenesis abundant) protein, its expression was higher at 10 DAA which is similar to reports in other crops like barley, where its expression was higher in late embryo maturation and during seed dehydration and played a vital role in the seed maturation process. Likewise, the level of granule-bound starch synthase-2 expressions also depends on the relative level of miR-166f and Cc_lncRNA-2117. In our study, it was observed that highest level of granule-bound starch synthase-2 expression occurs during 20 DAS, which reveals that during seed and pod development, source synthesized by the plant through photosynthesis was converted into starch for long term storage in the seed. In the case of maize, it was seen that there is a gradual increase in expression of granule-bound starch synthase-2 from 6 to 30 days after anthesis when the maximum accumulation of amylase occurs. In the case of *C*. *cajan* also, at 20 DAA, the accumulation of amylase was at higher levels in the endosperm. So overall study reveals that a complex lncRNA, miRNA, and mRNA interacting network is involved during the seed and pod developmental stages and all the interacting eTMs (lncRNAs) miRNAs and mRNAs interaction pattern is shown in Supplementary Fig. S4.

## Conclusion

Noncoding RNA is an essential element for gene regulation in plant and animal genomes. In our study we have predicted high fidelity 3019 lncRNAs and 227 miRNAs from the transcriptome data; It was observed that the lncRNA targets a group of mRNAs including TFs which are involved in various hormonal pathways required for seed and pod development, cell proliferation, cell division, signaling based pathways and genes involved in seed development and maturation. It was also observed that the predicted miRNAs also target the TFs like bHLH, MADS-box, NAC, etc. which are primarily involved in seed development, starch synthesis, sugar transporters, seed maturation, and fatty acid biosynthesis. More interestingly, we deduced that lncRNAs and miRNAs interact to form an eTMs, which ultimately regulate the mRNA expression. We validated our eTMs results via real-time- PCR analysis and observed that miRNAs expression was reduced when its corresponding eTMs, (the lncRNAs) sequesters them; as a result, the miRNA targets like sugar transporter, auxin-related F-box protein, starch synthetase, SPIKE genes were up-regulated during seed and pod development stages. Overall our analysis gives a clear insight that these ncRNAs (lncRNAs and miRNAs) interact with mRNAs and also with each other during the seed and pod development in *C*. *cajan*.

## Materials and Methods

### Transcriptome data and lncRNA identification pipeline

RNAseq data generated from three tissues and five different stages of seed and pod development (*C*. *cajan* var Asha) was used for identification of Cc_lncRNAs and miRNAs. The ten samples of tissues included leaf; flower bud at 0 days of anthesis (DAA); seed at five days after anthesis (DAS), 10 DAS, 20 DAS, and 30 DAS; pod at five days after anthesis (DAP), 10 DAP, 20 DAP, and 30 DAP. The sequence data of all the mentioned tissue and stages comprising NCBI accession number SRR4341972, SRR4341973, SRR4341974, SRR4341975, SRR4341976, SRR4341977, SRR4341978, SRR4341979, SRR4341980, and SRR054580 were downloaded from NCBI. Adaptor trimming and removal of low-quality reads was performed using Trimomatic 0.36 software with default parameters. Each dataset was mapped to the *C*. *cajan* genome through Tophat 2.0 program^34,35^, and all the dataset were assembled via the Cufflinks 2.0 program^35^, and Cuffmerge program from Cufflinks was used to merge all the pooled transcripts. Cuffdiff was performed with accepted BAM files (output file from Tophat 2.0) to calculate the abundance of the transcript.

### Prediction of lncRNAs

All the transcripts having strand information with the characteristics of non-overlap to known genes were taken for further analysis. Intergenic lncRNA (lincRNA), intragenic lncRNA, and natural antisense transcripts (lncNAT) were identified based on their location and strand information. The transcripts having FPKM (fragments per kilobase of transcript per million mapped reads) more than 1, and length above 200 bp were selected. ORF length was screened using ORF finder (http://www.ncbi.nlm.nih.gov/gorf/orfig.cgi) and transdecoder. The transcripts having <100 amino acid were further processed for their coding potential. Coding potential of the leftover transcripts was calculated using CPC^60^ and CNCI^61^ programs.

Transcripts having CPC and CNCI scores less than −0.5 were selected for further downstream analysis. Swiss-Prot database, NCBI non-redundant protein database, COGs databases, and KEGG protein database were used to perform BLASTX with remaining transcripts. The transcripts having considerable homology with known proteins were discarded. After that, all other groups of ncRNAs like tRNAs, rRNAs, tasiRNAs, snRNAs, miRNAs, and snoRNAs were discarded from the transcript data, and finally, lncRNAs were identified. All physical properties of lncRNAs were studied, and differentially expressed lncRNAs with log_2_fold was phased out from the cuffdiff results. DE pattern analysis and hierarchical clustering of lncRNAs was done through MeV 4.8.1 software by providing Cuffdiff result as input.

### Prediction of lncRNAs targeted mRNAs

Both *cis*-acting and *trans*-acting mRNA targets were identified for lncRNAs. The lncRNAs present in 10 kb window of upstream and downstream of the gene were considered as potential *cis-*target genes^62^ and mRNA having a complementary sequence to lncRNAs and coded by the genes which are not in the vicinity of lncRNA gene were considered as *trans*-acting targets. First, we used BLASTn to select target sequences that were complementary to the lncRNA, setting E-value ≤ 1e^−5^, and identity ≥95%. Then we used the RNAplex software to calculate the complementary energy between two sequences for further screening and to select potential *trans*-acting target genes (RNAplex dNG-50)^63^.

We calculated the Pearson correlation coefficient between the expression changes (in terms of log2 fold changes as a reference to zero days of anthesis) of lncRNAs and their target mRNAs (genes) in different stages of seed and pod development.

### miRNA prediction from the transcript data related to seed and pod development

All known miRNA and pre-miRNA were downloaded from miRBase21. Non-coding transcripts obtained from all the ten transcriptome data were used for miRNA prediction. The miRNA and pre-miRNA from miRBase21 was subjected to BLASTn against these pooled transcripts with the parameter (mismatch less than 3, word size 7 and e-value cut-off of 1000). To confirm its noncoding property again, Blastx was carried out against *C*. *cajan* protein database with the sequence identity cutoff ≥80% and all the protein-coding sequences fall in this cutoff was removed. Rest of the sequences were processed through CPC and CNCI with reference to the NR database, and the coding sequences were excluded.

All the non-coding transcripts were processed using the following parameters

(i) A Proper stem-loop hairpin secondary structure should be formed with minimum folding free energy and MFEI ≥ 0.41

(i) AU content should be 22–77% of the sequences
(ii) The mature miRNA sequence should be positioned in one arm of the hairpin structure
(iii) There should not be any loop or break in miRNA* sequence
(iv) miRNA sequence with opposite miRNA* should not have more than six mismatches
(v) Normalized Shannon entropy (NQ), Normalized base-pair distance (ND) and Normalized base-pairing propensity (Npb) value should be ≤ 0.45, ≤ 0.15 and ≥ 0.25, respectively
(xvi) SSR(simple sequence repeat) signature value R ≥ 2.5 with corresponding miRNA family

From the multiple BLAST hits, we chose only those sequences that follow all the parameter and having maximum MFEI and R-value.

### Prediction of miRNA targets

All the predicted miRNAs sequences were subjected to identify their target mRNA using psRNATarget software with default parameter^64^. To identify mRNAs as miRNAs target, mRNA sequences of *C*. *cajan* were used as targets query against miRNAs. Similarly, miRNAs as lncRNA target were identified using the same software, but the predicted miRNAs were used as the target query against predicted lncRNAs.

### Identification of lncRNAs as candidate endogenous target mimics

Endogenous target mimics (eTMs) is build-up for pairing among miRNAs and lncRNAs as it occurs in cells^65,66^. The eTMs among lncRNAs and miRNAs were discovered via the parameters enlisted below

(i) Only one bulge is allowed among miRNA and lncRNA, preferably 9^th^ to the 12^th^ nucleotide at 5′ end of miRNA.
(ii) The bulge present in eTMs should be only for three nucleotides; perfect nucleotide pairing essential at 2^nd^ to 8^th^ nucleotide at end of lncRNAs.
(iii) The total mismatches and G/U pairs within eTM and miRNA pairing regions should be less than three except for the central bulge. (Wu *et al*., 2012)^66^.

The psRobot software was used to identify the putative eTMs.

### Prediction and visualization of interaction among and between non-coding and coding RNAs

The secondary structures of lncRNAs and miRNAs were predicted with the Vienna RNA package RNAfold web (http://rna.tbi.univie.ac.at/). Identification and visualization of interaction network build between lncRNAs, miRNAs, and their target mRNA was done using Cytoscape (http://www.cytoscape.org/).

### Gene Ontology (GO) Annotation of lncRNAs, miRNA targeted mRNAs and lncRNA, targeted mRNAs

Gene Ontology annotation is an affirmation regarding the function of RNA or gene; this annotation program is always associating a RNA, or gene with a GO term. GO annotations confine about the gene functions and their involvement at the molecular level, cellular functions, and biological processes (pathways, programs). Molecular Function of RNAs reveals in which molecular activities they are involved. Cellular Component of RNAs interpret their location of availability, and the location where the RNAs and its products are active Biological Process interpret that the RNAs and their products are actively contributing the pathways and processes involved in the biological system. GO evidence code is supported each annotation. Annotation of lncRNAs, miRNA, mRNA targeted by miRNA, and mRNA targeted by lncRNAs was performed using Blast2Go analysis pipeline.

### RNA isolation, quantification, and cDNA synthesis

Total RNA was isolated from seven different tissue viz 0 DAA, 10 DAS, 20 DAS, 30 DAS, 10 DAP, 20 DAP, and 30 DAP using Spectrum Plant Total RNA Kit (SIGMA). RNase-free DNase (Ambion) treatment was given to 10 µg of total RNA to digest the unwanted genomic DNA. Nanodrop (ND-1000 spec) was used for quantification of isolated RNA. RNA integrity and quality were checked by electrophoresis of RNA on 1.2% denaturing agarose gel. The cDNA synthesis was performed using the Fermentas cDNA synthesis kit(Thermo fisher Scientific, Waltham, USA), as per manufacturer’s protocol.

### miRNA isolation, cDNA synthesis, and real-time PCR

The miRNA was isolated by protocol given in RNASure Fusion miRNA Minikit (Genetix) from all the tissue (0 DAA, 10 DAS, 10 DAP, 20 DAS, 20 DAP, 30 DAS and 30 DAP). Quantification of isolated mRNA was taken using Nanodrop 1000 spectrophotometer, and cDNA synthesis was carried out with MirX miRNA first-strand synthesis kit (Clontech, CA, USA).

### Validation of candidate lncRNAs, miRNAs and their target genes using quantitative real-time PCR

Prospective pigeon pea lncRNAs, miRNAs, and their target’s expression were analyzed using Light Cycler (Roche) real-time PCR instrument. All the primers required in the analysis were designed through IDT software (Integrated DNA Technologies, Inc., the US) (Supplementary S12). All the reaction was carried out in 96 well PCR plate. Brilliant III Ultra-Fast SYBR Green QPCR Master Mix kit (Agilent Technologies, USA) was used for qPCR analysis. 2 µl of cDNA, 0.5 µl of each one primer, 10 µl SYBR green (ROX added) and DEPC water were used per sample. The cDNA obtained from all the selected tissues and stages were used as a template for qRT-PCR analysis. The following reaction condition was set in Light Cycler (Roche) real-time PCR for amplification of templates. The initial denaturation temperature was 94 °C for 3 min, followed by 40 cycles of 94 °C for the 30 s, 60 °C for 15 s and 72 °C for 20 s. The melting curve study ranged from 56 to 95 °C, with increasing temperature steps of 0.5 °C every 10 s after completion of the amplification cycles. Tubulin-3α was used as an internal control for all the qRT-PCR reaction. Ubiquitin 6 (U6) was used as an endogenous control for all miRNAs. Three technical replicates were used for each sample. The specificity of the primers for each amplicon was checked by performing the melting curve analysis. The ‘comparative Ct method’ was used for the calculation of relative expression of each sample. The correlation between lncRNAs expression deliberate by RNAseq (FPKM) and qRT-PCR was analyzed via the R statistical package.

## Supplementary information

Supplementary Information

Supplementary S1

Supplementary S2

Supplementary S3

Supplementary S4

Supplementary S5

Supplementary S6

Supplementary S7

Supplementary S8

Supplementary S9

Supplementary S10
