# Supplementary material for: Expressivity of the key genes associated with seed and pod development is highly regulated via lncRNAs and miRNAs in Pigeonpea
Source: Sci Rep. 2019 Dec 3;9:18191. doi: 10.1038/s41598-019-54340-6 (PMC6890743; doi:10.1038/s41598-019-54340-6)
Supplement: Supplementary file 1 — Supplementary Information [file 41598_2019_54340_MOESM1_ESM.pdf]

Supplementary Information:

Expressivity of the key genes associated with seed and pod development is highly regulated via lncRNAs and miRNAs in Pigeonpea

Antara Das[1], Deepti Nigam[1], Alim Junaid[1], Kishor U Tribhuvan[1], Kuldeep Kumar[1], Kumar Durgesh[2], Singh NK[1], Kishor Gaikwad[1]*

1 ICAR- National Research Centre on Plant Biotechnology, New Delhi, India

2 Division of Genetics, ICAR-IARI, New Delhi, India

*Corresponding author's email ID: kish2012@nrcpb.org, kish2012@gmail.com

Supplementary data:

Supplementary Figure 1. Gene Ontology (GO) analysis of predicted lncRNAs; BP- Biological Process, MF- Molecular Function, CC- Cellular Component.

Supplementary Figure 2. miRNA and mRNA interacting network, yellow circles represent miRNAs, and orange circles represent mRNAs

Supplementary Figure 3. miRNA and lncRNA interacting network via eTM*s*, yellow circles represent miRNAs, and red circles represent lncRNAs

Supplementary Figure 4. lncRNA, miRNA, and mRNA interacting network, yellow circles represents miRNAs, and red circles represent lncRNAs and mRNAs

Supplementary S1. Transcripts read information

Supplementary S2. All physical properties of predicted lncRNAs

Supplementary S3. Differentially expressed lncRNAs and their log fold change value

Supplementary S4. Targets of lncRNAs (lncRNAs with their corresponding target mRNAs with Dng value, Pearson correlation coefficient result for interacting lncRNA, and the target mRNA, all mRNAs and TFs information targeted by mRNAs)

Supplementary S5. Gene Ontology (GO) distribution of predicted lncRNAs

Supplementary S6. Gene Ontology (GO) distribution of predicted mRNAs targets of lncRNAs

Supplementary S7. Physical properties of identified miRNAs

Supplementary S8. miR-family wise distribution of predicted miRNAs

Supplementary S9. MiRNA Targets including TFs (mRNA's UTRs targeted by miRNAs, all mRNAs and TFs targeted by mRNAs)

Supplementary S10. Gene Ontology (GO) analysis of mRNAs targeted by miRNAs

Supplementary S11. lncRNAs act as Endogenous target mimics (eTMs) with corresponding miRNAs with their pairing information.

Supplementary S12. Primer name and sequences used in this study.

Supplementary Figures:

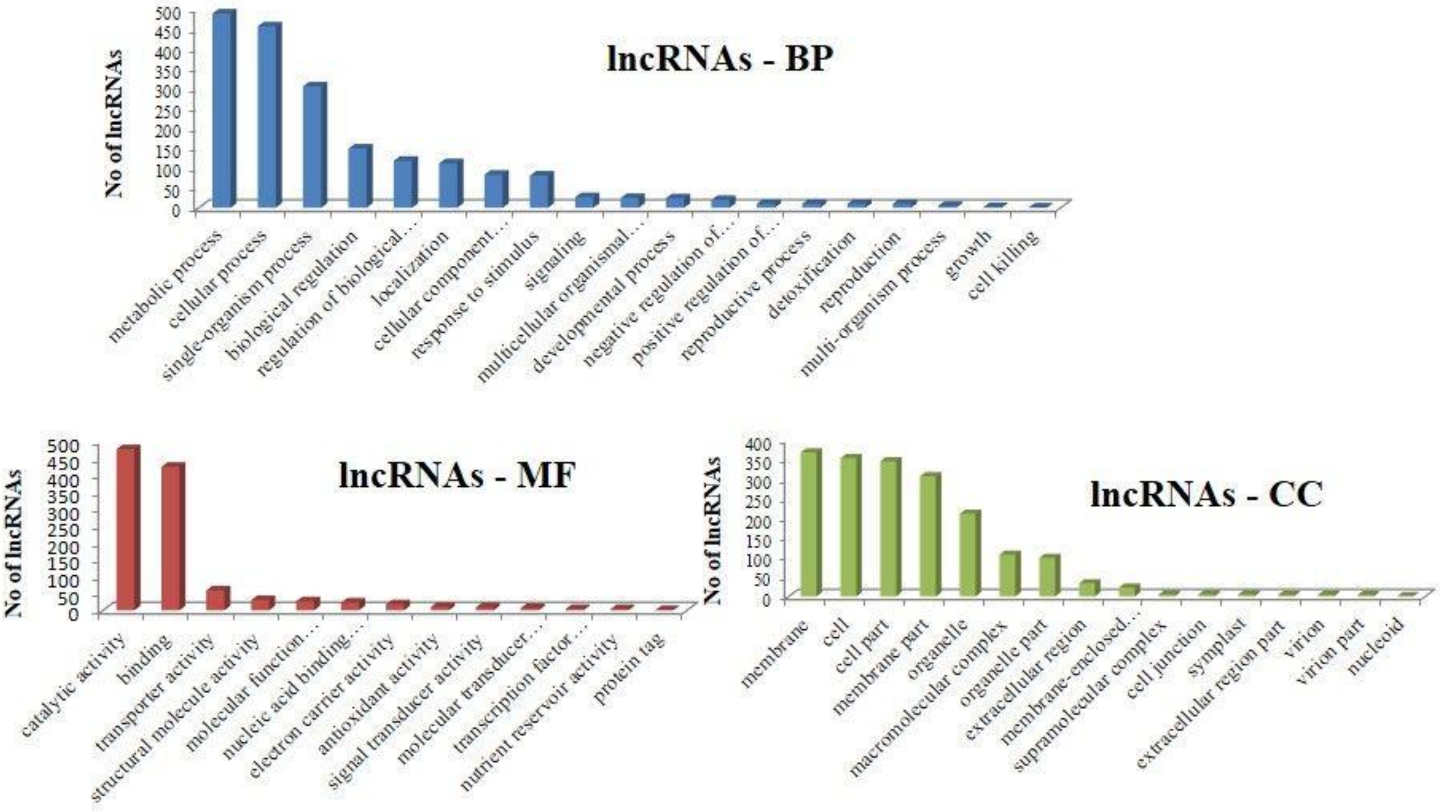

Supplementary Figure 1. Gene Ontology (GO) analysis of predicted lncRNAs; BP- Biological Process, MF- Molecular Function, CC- Cellular Component.

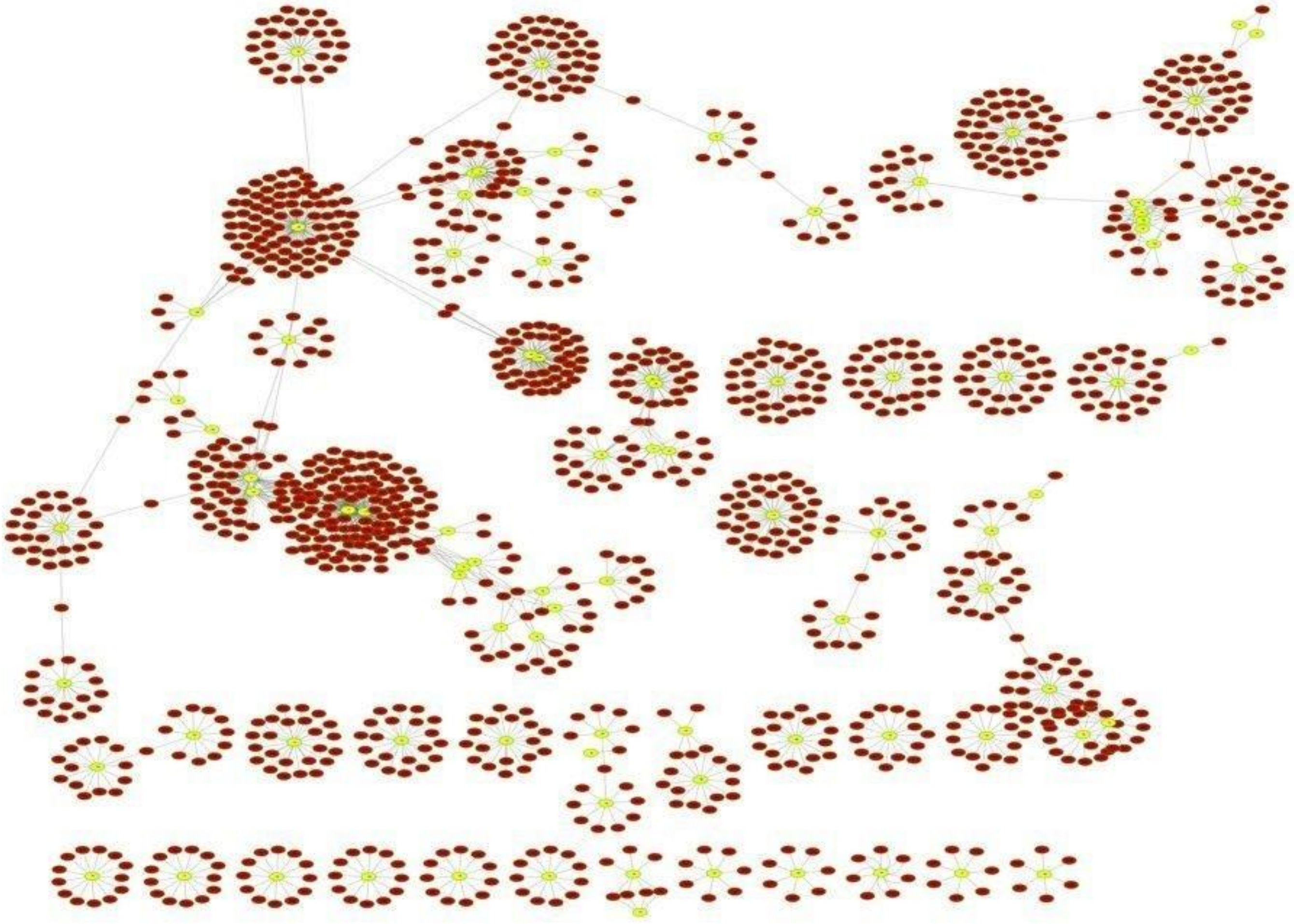

Supplementary Figure 2. miRNA and mRNA interacting network, yellow circles represent miRNAs, and orange circles represent mRNAs

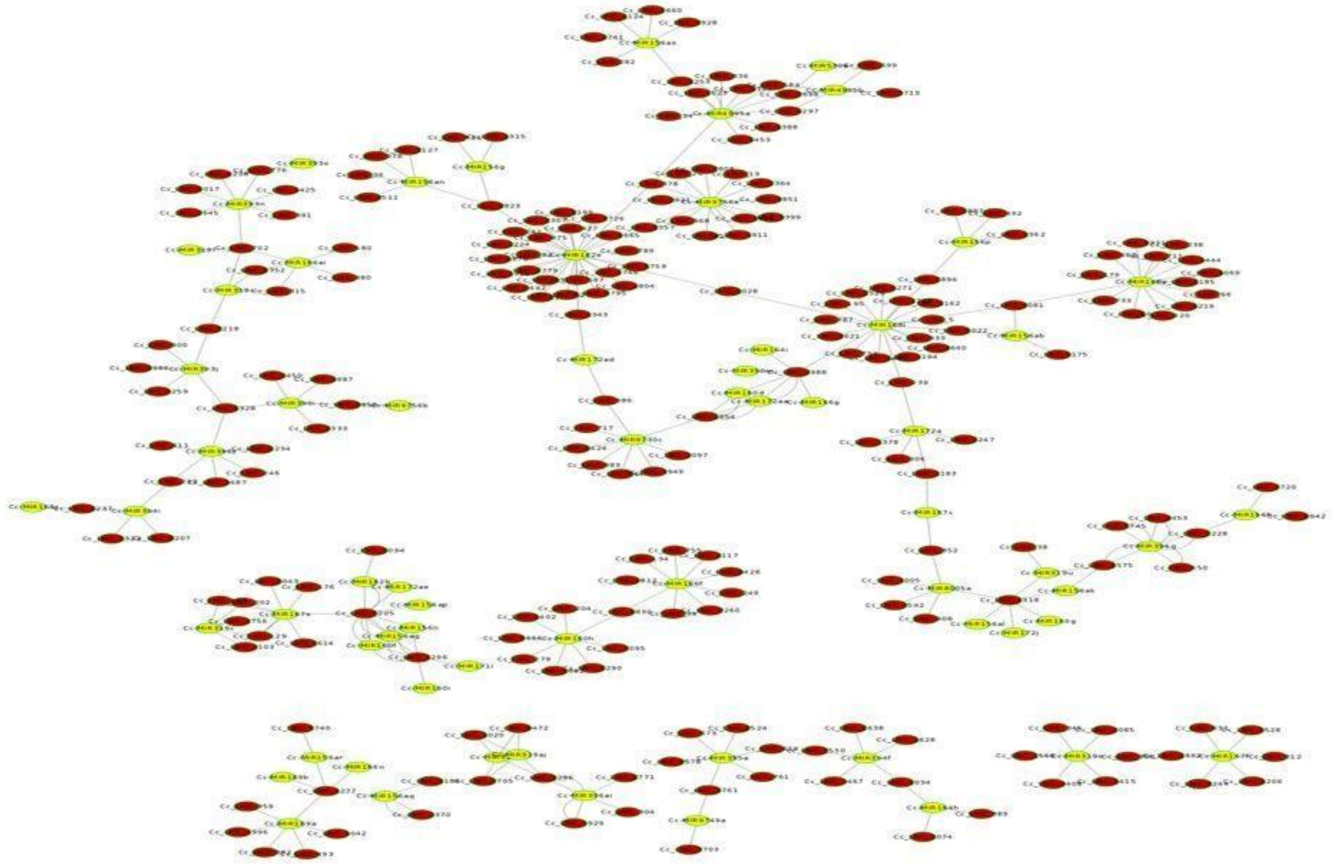

Supplementary Figure 3. miRNA and lncRNA interacting network via eTM*s*, yellow circles represent miRNAs, and red circles represent lncRNAs

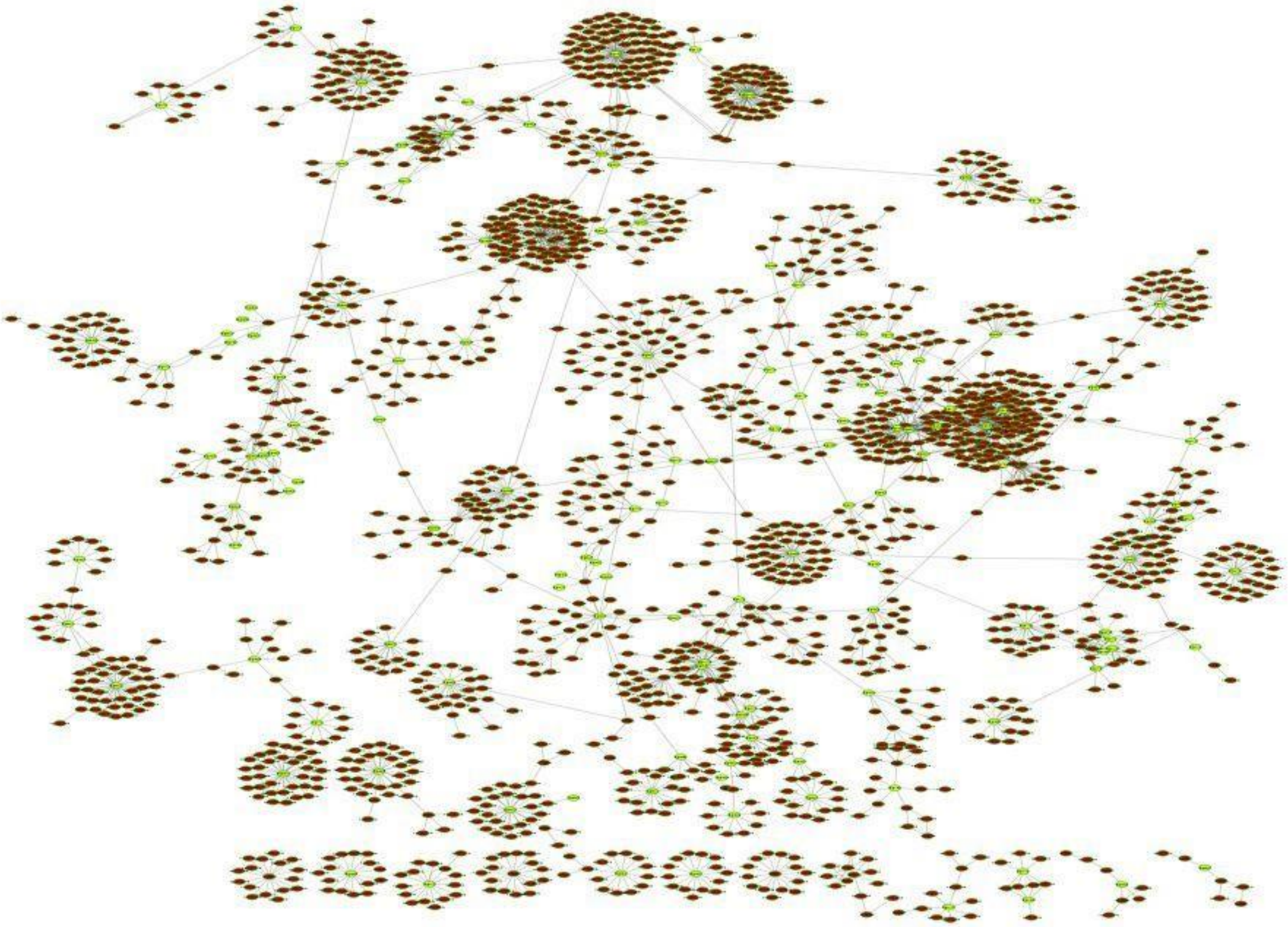

Supplementary Figure 4. lncRNA, miRNA, and mRNA interacting network, yellow circles represents miRNAs, and red circles represent lncRNAs and mRNAs

Supplementary S11. lncRNAs act as Endogenous target mimics (eTMs) with corresponding miRNAs with their pairing information.

```
 miRNA(Query)        lncRNA(Sbjct)              Score                   Alignment
                                                      Query:        1 AAAATATTTGTACATGATT 19
                                                                      ||||||||| ||||||||:
Cc_miR-1310c    KQ483458.1:313869-318266          1.5 Sbjct:     2282 TTTTATAAAAATGTACTAG 2264
                                                      Query:        1 AAAATATTTGTACATGATT 19
                                                                      |||||||||||| | ||||
Cc_miR-1310c    KQ483541.1:146789-151565            2 Sbjct:      471 TTTTATAAACATTTTCTAA 453
                                                      Query:        1 AAAATATTTGTACATGATT 19
                                                                      | ||||:|:||||||||||
Cc_miR-1310c    KQ485114.1:29686-30328              2 Sbjct:       33 TATTATGAGCATGTACTAA 15
                                                      Query:        1 AAAGTATGAGAGGGAGA 17
                                                                      |||||||||:||::|:|
Cc_miR-1310f    CM003609.1:14614858-14619325        2 Sbjct:     2367 TTTCATACTTTCTTTTT 2351
                                                      Query:        1 AAAGTATGAGAGGGAGA 17
                                                                      |||:||||||||::|:|
Cc_miR-1310f    KQ483710.1:78340-81792              2 Sbjct:     2505 TTTTATACTCTCTTTTT 2489
                                                      Query:        1 AAAGTATGAGAGGGAGA 17
                                                                      || |||:||||||:|||
Cc_miR-1310f    KQ484027.1:110292-120971            2 Sbjct:     9521 TTACATGCTCTCCTTCT 9505
                                                      Query:        1 AAATCCAAAGGGAT 14
                                                                      |||||||||:|:||
Cc_miR-1507b    KQ483545.1:105340-105630            1 Sbjct:      155 TTTAGGTTTTCTTA 142
                                                      Query:        1 AAATCCAAAGGGAT 14
                                                                      |||||||||:|:||
Cc_miR-1507b    KQ483712.1:172309-180051            1 Sbjct:     5336 TTTAGGTTTTCTTA 5323
                                                      Query:        1 AAATCCAAAGGGAT 14
                                                                      |||||||||||||
Cc_miR-1507b    KQ485289.1:10466-10850              1 Sbjct:      128 TTTAGGTTTCCCTT 115
```

```
                                                          Query:         1 AGAAAAATATGAAGTAAAAATGAAATAA 28
                                                                           ||||||||| |||| |||||||||||||
Cc_miR-156ab    KQ483558.1:178490-181243              2   Sbjct:      1068 TCTTTTTATCCTTCCTTTTTACTTTATT 1041
                                                          Query:         1 AGAAAAATATGAAGTAAAAATGAAATAA 28
                                                                           |:||||||||:||: |||||||||||||
Cc_miR-156ab    CM003609.1:2794127-2798852          2.5   Sbjct:      2283 TTTTTTTATATTTTCTTTTTACTTTATT 2256
                                                          Query:         1 AGAAAAATATGAAGTAAAAATGAAATAA 28
                                                                           |||||||||::||: |||||||||||||
Cc_miR-156ab    CM003610.1:7414154-7415237          2.5   Sbjct:       814 TCTTTTTATGTTTTCTTTTTACTTTATT 787
                                                          Query:         1 AGAGATTAGACAATTGAATAGTAT 24
                                                                           ||||||||||||||||||||||||
Cc_miR-156ag    CM003611.1:4192668-4202075            0   Sbjct:      5438 TCTCTAATCTGTTAACTTATCATA 5415
                                                          Query:         1 AGAGATTAGACAATTGAATAGTAT 24
                                                                           ||||||||||||||||||||||||
Cc_miR-156ag    CM003611.1:4192668-4202075            0   Sbjct:      5128 TCTCTAATCTGTTAACTTATCATA 5105
                                                          Query:         1 AGAGATTAGACAATTGAATAGTAT 24
                                                                           ||||||||| ||||||||||||||
Cc_miR-156ag    CM003612.1:20929167-20941402          1   Sbjct:      5899 TCTCTAATCCGTTAACTTATCATA 5876
                                                          Query:         1 AGAGATTAGACAATTGAATAGTAT 24
                                                                           ||||||||| ||||||||||||||
Cc_miR-156ag    CM003612.1:20929167-20941402          1   Sbjct:      5515 TCTCTAATCCGTTAACTTATCATA 5492
                                                          Query:         1 AGAGGCTGACAGAAGAGAG 19
                                                                           |||::||||||:|||||||
Cc_miR-156aj    CM003607.1:627553-630800            1.5   Sbjct:      3224 TCTTTGACTGTTTTCTCTC 3206
                                                          Query:         1 AGAGTGAGCACACATGGC 18
                                                                           ||||||||||||||||||
Cc_miR-156ak    CM003612.1:3998296-3999928            1   Sbjct:      1423 TCTCACTCGTGTGTACCA 1406
                                                          Query:         1 AGAGTGAGCACACATGGC 18
                                                                           ||||||||||| |||::|
Cc_miR-156ak    KQ483994.1:22970-25497                2   Sbjct:       466 TCTCACTCGTGCGTATTG 449
                                                          Query:         1 AGAGTGAGCACACATGGCACTTTCT 25
                                                                           ||||||||||||||||| |||||||
Cc_miR-156al    CM003612.1:3998296-3999928            1   Sbjct:      1423 TCTCACTCGTGTGTACCATGAAAGA 1399
```

```
                                                 Query:        1 AGATGATAGCTTAAGG 16
                                                                  |||||||||||||:|
Cc_miR-156an   AGCT01021417.1:2658-6833     1.5  Sbjct:     1479 GCTACTATCGAATTTC 1464

                                                 Query:        1 AGATGATAGCTTAAGG 16
                                                                 ||||||||:||:||::
Cc_miR-156an   CM003609.1:13316047-13319607   2  Sbjct:     2060 TCTACTATTGAGTTTT 2045

                                                 Query:        1 AGATGATAGCTTAAGG 16
                                                                  :||||||||||||:|
Cc_miR-156an   KQ483582.1:284978-286006       2  Sbjct:      885 GTTACTATCGAATTTC 870

                                                 Query:        1 AGATGATAGCTTAAGG 16
                                                                 |||::|||| ||||||
Cc_miR-156an   KQ483894.1:145398-154587       2  Sbjct:     1366 TCTGTTATCAAATTCC 1351

                                                 Query:        1 AGATGATAGCTTAAGG 16
                                                                 ||||||:|:|| ||||
Cc_miR-156an   KQ484729.1:14909-32754         2  Sbjct:    10739 TCTACTGTTGATTTCC 10724

                                                 Query:        1 AGCATAAGCCATATATATT 19
                                                                 || ||||||||| ||:|||
Cc_miR-156ap   CM003611.1:4192668-4202075   2.5  Sbjct:     6959 TCCTATTCGGTACATGTAA 6941

                                                 Query:        1 AGCATCATCAAGATTC 16
                                                                 |||| |||||||||||
Cc_miR-156aq   CM003612.1:1832435-1832939     1  Sbjct:       58 TCGTCGTAGTTCTAAG 43

                                                 Query:        1 AGCATCATCAAGATTC 16
                                                                 |:|||||:|||||:||
Cc_miR-156aq   CM003610.1:9153116-9155558   1.5  Sbjct:      452 TTGTAGTGGTTCTGAG 437

                                                 Query:        1 AGCATCATCAAGATTC 16
                                                                 | |||||||||:||||
Cc_miR-156aq   KQ483749.1:133629-135061     1.5  Sbjct:      800 TGGTAGTAGTTTTAAG 785

                                                 Query:        1 AGCATCATCAAGATTC 16
                                                                 |:|||||:|||:||||
Cc_miR-156aq   KQ483749.1:133629-135061     1.5  Sbjct:      460 TTGTAGTGGTTTTAAG 445

                                                 Query:        1 AGCATCATCAAGATTCTCA 19
                                                                 |||| ||||||||||| ||
Cc_miR-156ar   CM003612.1:1832435-1832939     2  Sbjct:       58 TCGTCGTAGTTCTAAGTGT 40
```

```
                                                    Query:         1 AGCATCATCAAGATTCTCA 19
                                                                     ||||||||||| |::||||
Cc_miR-156ar   KQ483437.1:463791-465154         2   Sbjct:       839 TCGTAGTAGTTGTGGGAGT 821

                                                    Query:         1 AGCATGATCCCAAA 14
                                                                     |||||||||| |||
Cc_miR-156as   KQ485416.1:21308-22916           1   Sbjct:       184 TCGTACTAGGATTT 171

                                                    Query:         1 AGCATGATCCCAAA 14
                                                                     |||||| :||||||
Cc_miR-156as   CM003603.1:4458886-4461281     1.5   Sbjct:      2332 TCGTACGGGGGTTT 2319

                                                    Query:         1 AGCATGATCCCAAA 14
                                                                     |:|||||| |||||
Cc_miR-156as   CM003610.1:19178691-19180172   1.5   Sbjct:        99 TTGTACTATGGTTT 86

                                                    Query:         1 AGCATGATCCCAAA 14
                                                                     |:||||||| ||||
Cc_miR-156as   CM003612.1:12363471-12380411   1.5   Sbjct:      6259 TTGTACTAGTGTTT 6246

                                                    Query:         1 AGCATGATCCCAAA 14
                                                                     || ||||:||||||
Cc_miR-156as   KQ483418.1:977916-985074       1.5   Sbjct:       862 TCATACTGGGGTTT 849

                                                    Query:         1 AGCATGATCCCAAA 14
                                                                     |||| :||||||||
Cc_miR-156as   KQ484398.1:66426-67932         1.5   Sbjct:       396 TCGTTTTAGGGTTT 383

                                                    Query:         1 AATATTGTCGTTATTGTAATTC 22
                                                                     ||| |||||||:||||||||||
Cc_miR-156d    KQ483787.1:56661-58800         1.5   Sbjct:      1543 TTACAACAGCAGTAACATTAAG 1522

                                                    Query:         1 AATGGAGGAATTTGAATTA 19
                                                                     | ||||:||||||||||||
Cc_miR-156g    CM003604.1:26845381-26850877   2.5   Sbjct:       251 TCACCTTCTTAAACTTAAA 233

                                                    Query:         1 AATGGAGGAATTTGAATTA 19
                                                                     ||| |||:||||||| |||
Cc_miR-156g    CM003612.1:3657054-3661951     2.5   Sbjct:      2896 TTAACTCTTTAAACTGAAT 2878

                                                    Query:         1 AATGGAGGAATTTGAATTA 19
                                                                     |||::|::|||||:|||||
Cc_miR-156g    KQ484729.1:14909-32754         2.5   Sbjct:      2228 TTATTTTTTTAAATTTAAT 2210
```

```
                                                        Query:     1 ACAACCCTCACTTTACAAGCCGGTTT 26
                                                                      ||||||||||:||||||||||||||
Cc_miR-156n   CM003611.1:4192668-4202075          1.5  Sbjct:  5093 CGTTGGGAGTGGAATGTTCGGCCAAA 5068
                                                        Query:     1 ACAACCCTCACTTTACAAGCCGGTTT 26
                                                                      ||||||||||:||||||||||||||
Cc_miR-156n   CM003612.1:20929167-20941402        1.5  Sbjct:  5864 CGTTGGGAGTGGAATGTTCGGCCAAA 5839
                                                        Query:     1 ACAACCCTCACTTTACAAGCCGGTTT 26
                                                                      ||||||||||:||||||||||||||
Cc_miR-156n   CM003612.1:20929167-20941402        1.5  Sbjct:  5480 CGTTGGGAGTGGAATGTTCGGCCAAA 5455
                                                        Query:     1 ACAACCCTCACTTTACAAGCCGGTTT 26
                                                                      ||||||| ||:||||||||||||||
Cc_miR-156n   CM003611.1:4192668-4202075          2.5  Sbjct:  5403 CGTTGGGAATGGAATGTTCGGCCAAA 5378
                                                        Query:     1 ACACTTCTAGGAGAGAAAAT 20
                                                                     ||||||||| ||||||||||
Cc_miR-156p   KQ483482.1:180801-185551              1  Sbjct:  3982 TGTGAAGATACTCTCTTTTA 3963
                                                        Query:     1 ACACTTCTAGGAGAGAAAAT 20
                                                                     |||||||||:||||:|||||
Cc_miR-156p   KQ483485.1:242489-245051              1  Sbjct:  1378 TGTGAAGATTCTCTTTTTTA 1359
                                                        Query:     1 ACACTTCTAGGAGAGAAAAT 20
                                                                      ||||| |||||||||||||
Cc_miR-156p   CM003604.1:3826881-3830549            2  Sbjct:  2812 CGTGAAAATCCTCTCTTTTA 2793
                                                        Query:     1 ACACTTCTAGGAGAGAAAAT 20
                                                                     ||| |||||||||||||||
Cc_miR-156p   CM003613.1:10808887-10816294          2  Sbjct:  6026 TGTAAAGATCCTCTCTTTTT 6007
                                                        Query:     1 ACTTGTAGGCGTTTT 15
                                                                     | |||||:|||||||
Cc_miR-156z   AGCT01045937.1:123-2142             1.5  Sbjct:   141 TAAACATTCGCAAAA 127
                                                        Query:     1 AGGATAGGCGGGACTTGGGTTTTTCCCAAT 30
                                                                     |||||||||::|||||||||||||||||||
Cc_miR-160d   KQ483469.1:394906-406708              1  Sbjct:  4414 TCCTATCCGTTCTGAACCCAAAAAGGGTTA 4385
                                                        Query:     1 AGGATAGGCGGGACTTGGGTTTTTCCCAAT 30
                                                                     |||||||||:|||||||||||||||||  |
Cc_miR-160d   KQ483469.1:394906-406708            2.5  Sbjct:  4456 TCCTATCCGTCCTGAACCCAAAAAGGGGAA 4427
```

```
                                                           Query:         1 AGGATAGGCGGGACTTGGGTTTTTCCCAAT 30
                                                                            ||||| |:|:||||||:|||||||||||||
Cc_miR-160d      KQ486010.1:2815-10076                2.5  Sbjct:      3099 TCCTAACTGTCCTGAATCCAAAAAGGGTTA 3070

                                                           Query:         1 AGGGGGGTGTATTGGGAAAAACCCAAGTCC 30
                                                                            |||||||||||||||:||||||||||||||
Cc_miR-160f      CM003611.1:4192668-4202075           0.5  Sbjct:      5168 TCCCCCCACATAACCTTTTTTGGGTTCAGG 5139

                                                           Query:         1 AGGGGGGTGTATTGGGAAAAACCCAAGTCC 30
                                                                             :||||||||||||||||||||||||||||
Cc_miR-160f      CM003611.1:4192668-4202075           1.5  Sbjct:      5270 CTCCCCCACATAACCCTTTTTGGGTTCAGG 5241

                                                           Query:         1 AGGGGGGTGTATTGGGAAAAACCCAAGTCC 30
                                                                             :||||||||||||||||||||||||||||
Cc_miR-160f      CM003612.1:20929167-20941402         1.5  Sbjct:      5939 CTCCCCCACATAACCCTTTTTGGGTTCAGG 5910

                                                           Query:         1 AGGGGGGTGTATTGGGAAAAACCCAAGTCC 30
                                                                             :||||||||||||||||||||||||||||
Cc_miR-160f      CM003612.1:20929167-20941402         1.5  Sbjct:      5555 CTCCCCCACATAACCCTTTTTGGGTTCAGG 5526

                                                           Query:         1 AGGTGACAGAAGAGAGTGAGCACACATGGC 30
                                                                            |||||||||| ||||||||||||||||||
Cc_miR-160g      CM003612.1:3998296-3999928             2  Sbjct:      1435 TCCACTGTCTGCTCTCACTCGTGTGTACCA 1406

                                                           Query:         1 AGGTTAAGGTGGAGAT 16
                                                                            | ||||||||||||||
Cc_miR-160h      KQ484745.1:39350-42640                 1  Sbjct:      2109 TACAATTCCACCTCTA 2094

                                                           Query:         1 AGGTTAAGGTGGAGAT 16
                                                                            ||||||||: |||||:
Cc_miR-160h      AGCT01068337.1:15165-19202             2  Sbjct:      2691 TCCAATTCTCCCTCTG 2676

                                                           Query:         1 AGGTTAAGGTGGAGAT 16
                                                                            ||:|||||||| |:||
Cc_miR-160h      CM003603.1:3666913-3671325             2  Sbjct:      2405 TCTAATTCCACGTTTA 2390

                                                           Query:         1 AGGTTAAGGTGGAGAT 16
                                                                            |||| |||||||| ||
Cc_miR-160h      CM003612.1:20835884-20844274           2  Sbjct:      5952 TCCACTTCCACCTATA 5937

                                                           Query:         1 AGGTTAAGGTGGAGAT 16
                                                                            ||||||||:|::|:||
Cc_miR-160h      CM003613.1:18529325-18531651           2  Sbjct:       962 TCCAATTCTATTTTTA 947
```

```
                                                    Query:     1 AGGTTAAGGTGGAGAT 16
                                                                 ||||||||| |:|:||
Cc_miR-160h   KQ483424.1:582589-585095          2   Sbjct:  1215 TCCAATTCCCCTTTTA 1200

                                                    Query:     1 AGGTTAAGGTGGAGAT 16
                                                                 |||||||:: ||||||
Cc_miR-160h   KQ483561.1:292215-294724          2   Sbjct:   648 TCCAATTTTCCCTCTA 633

                                                    Query:     1 AGGTTAAGGTGGAGAT 16
                                                                 |::|||||:||:||||
Cc_miR-160h   KQ484145.1:84132-84659            2   Sbjct:   468 TTTAATTCTACTTCTA 453

                                                    Query:     1 AGTTAGGCTTAATCCACTATTCTAACATGA 30
                                                                 ||||||||||||||||||||||||||||||
Cc_miR-160i   CM003612.1:20929167-20941402      1   Sbjct:  5828 TCAATCCGAATTAGGTGATAAGATTGTACC 5799

                                                    Query:     1 AGTATATGAGAAAGAATTAATG 22
                                                                 |:| |||||||||:|||||||:
Cc_miR-160m   CM003613.1:14685672-14692067    2.5   Sbjct:   875 TTAAATACTCTTTTTTAATTAT 854

                                                    Query:     1 ATATTGAAAAAAATCCAAGTC 21
                                                                  ||||| ||||||:|||||||
Cc_miR-162b   CM003611.1:4192668-4202075      2.5   Sbjct:  5470 GATAACCTTTTTTGGGTTCAG 5450

                                                    Query:     1 ATATTGAAAAAAATCCAAGTC 21
                                                                  ||||| ||||||:|||||||
Cc_miR-162b   CM003611.1:4192668-4202075      2.5   Sbjct:  5160 CATAACCTTTTTTGGGTTCAG 5140

                                                    Query:     1 ATATTGAAAAAAATCCAAGTC 21
                                                                 |||||| ||||||| |||:||
Cc_miR-162b   KQ483561.1:210811-211853        2.5   Sbjct:   575 TATAACCTTTTTTAAGTTTAG 555

                                                    Query:     1 ATTTGTTATTTATTTATT 18
                                                                 |||| |||||||||||||
Cc_miR-162e   CM003610.1:20083071-20090945      1   Sbjct:  5865 TAAAAAATAAATAAATAA 5848

                                                    Query:     1 ATTTGTTATTTATTTATT 18
                                                                 ||||:|||||||||:|||
Cc_miR-162e   CM003613.1:47138515-47139566      1   Sbjct:   211 TAAATAATAAATAAGTAA 194

                                                    Query:     1 ATTTGTTATTTATTTATT 18
                                                                 |||| |||||||||||||
Cc_miR-162e   KQ483452.1:621686-623630          1   Sbjct:   419 TAAAAAATAAATAAATAA 402
```

```
                                                         Query:      1 ATTTGTTATTTATTTATT 18
                                                                       ||||||:||:||||||||
Cc_miR-162e    KQ483735.1:15946-19585                 1  Sbjct:   2482 TAAACAGTAGATAAATAA 2465

                                                         Query:      1 ATTTGTTATTTATTTATT 18
                                                                       |||| |||||||||||||
Cc_miR-162e    KQ484509.1:35577-43013                 1  Sbjct:   4707 TAAAAAATAAATAAATAA 4690

                                                         Query:      1 ATTTGTTATTTATTTATT 18
                                                                       |||| |||||||||||||
Cc_miR-162e    KQ484729.1:14909-32754                 1  Sbjct:  15230 TAAAAAATAAATAAATAA 15213

                                                         Query:      1 ATTTGTTATTTATTTATT 18
                                                                        |||||||||||||||||
Cc_miR-162e    KQ484980.1:29270-36393                 1  Sbjct:   6453 AAAACAATAAATAAATAA 6436

                                                         Query:      1 ATTTGTTATTTATTTATT 18
                                                                       || |:|||||||||||||
Cc_miR-162e    AGCT01038449.1:0-203                 1.5  Sbjct:     73 TATATAATAAATAAATAA 56

                                                         Query:      1 ATTTGTTATTTATTTATT 18
                                                                        |||:|||||||||||||
Cc_miR-162e    CM003603.1:13172038-13173155         1.5  Sbjct:    115 AAAATAATAAATAAATAA 98

                                                         Query:      1 ATTTGTTATTTATTTATT 18
                                                                       |:|| |||||||||||||
Cc_miR-162e    CM003604.1:21520036-21526240         1.5  Sbjct:   4037 TGAAAAATAAATAAATAA 4020

                                                         Query:      1 ATTTGTTATTTATTTATT 18
                                                                       |:|| |||||||||||||
Cc_miR-162e    CM003604.1:28130812-28138510         1.5  Sbjct:   6886 TGAAAAATAAATAAATAA 6869

                                                         Query:      1 ATTTGTTATTTATTTATT 18
                                                                       ||||||| |||||||||:
Cc_miR-162e    CM003604.1:34615889-34617239         1.5  Sbjct:   1037 TAAACAAAAAATAAATAG 1020

                                                         Query:      1 ATTTGTTATTTATTTATT 18
                                                                       |||| |:||||||||||||
Cc_miR-162e    CM003605.1:16889780-16898678         1.5  Sbjct:   4349 TAAAAAGTAAATAAATAA 4332

                                                         Query:      1 ATTTGTTATTTATTTATT 18
                                                                       || |:|||||||||||||
Cc_miR-162e    CM003607.1:1710559-1713892           1.5  Sbjct:   2107 TATATAATAAATAAATAA 2090
```

```
                                                              Query:       1 ATTTGTTATTTATTTATT 18
                                                                             ||||:| |||||||||||
Cc_miR-162e      CM003609.1:3506223-3510535             1.5  Sbjct:    3021 TAAATATTAAATAAATAA 3004
                                                              Query:       1 ATTTGTTATTTATTTATT 18
                                                                             |||| |||||:|||||||
Cc_miR-162e      CM003611.1:296547-298649               1.5  Sbjct:    1193 TAAAAAATAAGTAAATAA 1176
                                                              Query:       1 ATTTGTTATTTATTTATT 18
                                                                             | :|||||||||||||||
Cc_miR-162e      CM003611.1:7807531-7817211             1.5  Sbjct:     335 TCGACAATAAATAAATAA 318
                                                              Query:       1 ATTTGTTATTTATTTATT 18
                                                                             ||||:|||||||||| ||
Cc_miR-162e      CM003612.1:14496005-14499639           1.5  Sbjct:    2442 TAAATAATAAATAAAAAA 2425
                                                              Query:       1 ATTTGTTATTTATTTATT 18
                                                                             ||||:|| ||||||||||
Cc_miR-162e      CM003612.1:3493333-3494440             1.5  Sbjct:     420 TAAATAAAAAATAAATAA 403
                                                              Query:       1 ATTTGTTATTTATTTATT 18
                                                                              |:|||||||||||||||
Cc_miR-162e      CM003612.1:7569934-7572859             1.5  Sbjct:     186 AAGACAATAAATAAATAA 169
                                                              Query:       1 ATTTGTTATTTATTTATT 18
                                                                             |:|| |||||||||||||
Cc_miR-162e      CM003612.1:855866-867157               1.5  Sbjct:    9845 TGAAAAATAAATAAATAA 9828
                                                              Query:       1 ATTTGTTATTTATTTATT 18
                                                                             ||||:|| ||||||||||
Cc_miR-162e      KQ483438.1:511856-513118               1.5  Sbjct:     326 TAAATAAAAAATAAATAA 309
                                                              Query:       1 ATTTGTTATTTATTTATT 18
                                                                             |:|| |||||||||||||
Cc_miR-162e      KQ483441.1:1158-2353                   1.5  Sbjct:      37 TGAAAAATAAATAAATAA 20
                                                              Query:       1 ATTTGTTATTTATTTATT 18
                                                                             || |||||||:|||||||
Cc_miR-162e      KQ483697.1:145204-150282               1.5  Sbjct:    3524 TACACAATAAGTAAATAA 3507
                                                              Query:       1 ATTTGTTATTTATTTATT 18
                                                                             ||:|:|||||||:|||||
Cc_miR-162e      KQ483824.1:142489-144148               1.5  Sbjct:      57 TAGATAATAAATGAATAA 40
```

```
                                                        Query:        1 ATTTGTTATTTATTTATT 18
                                                                        ||||||||||| ||||:|
Cc_miR-162e     KQ484342.1:79062-79543             1.5  Sbjct:      481 TAAACAATAAAGAAATGA 464
                                                        Query:        1 ATTTGTTATTTATTTATT 18
                                                                        |||: |||||||||||||
Cc_miR-162e     KQ484561.1:60744-63276             1.5  Sbjct:     1621 TAAGAAATAAATAAATAA 1604
                                                        Query:        1 ATCATCAAGATTCTCA 16
                                                                        ||||||||:|:|||||
Cc_miR-164h     CM003609.1:410339-419438             1  Sbjct:     2433 TAGTAGTTTTGAGAGT 2418
                                                        Query:        1 ATCATCAAGATTCTCA 16
                                                                        ||||:||| |||||||
Cc_miR-164h     CM003608.1:22127682-22128805       1.5  Sbjct:     1018 TAGTGGTTGTAAGAGT 1003
                                                        Query:        1 ATCATCAAGATTCTCA 16
                                                                        |:||| ||||||||||
Cc_miR-164h     KQ483555.1:165176-166301           1.5  Sbjct:     1026 TGGTAATTCTAAGAGT 1011
                                                        Query:        1 ATCCAAAGGGATCGCATTGATCCTA 25
                                                                        |||||||||||||||||||||||:|
Cc_miR-164i     KQ486010.1:2815-10076              0.5  Sbjct:     6812 TAGGTTTCCCTAGCGTAACTAGGGT 6788
                                                        Query:        1 ATCTACTAATTACTCCTT 18
                                                                        |: | |||||||||||||
Cc_miR-164l     KQ483538.1:218276-221634           2.5  Sbjct:     2952 TGAACGATTAATGAGGAA 2935
                                                        Query:        1 CCATGCCCTCTTTTCTTT 18
                                                                        |||||||||||||| ||
Cc_miR-166ad    KQ483798.1:18384-18976               2  Sbjct:      183 GGTACGGGAGAAAATAAC 166
                                                        Query:        1 CCATGCCCTCTTTTCTTT 18
                                                                        | |::|||||||||||||
Cc_miR-166ad    KQ484746.1:3009-6625                 2  Sbjct:     3234 GTTGTGGGAGAAAAGAAA 3217
                                                        Query:        1 CCTTTTTTTCACTTCTTA 18
                                                                        ||||||:||| |:|||||
Cc_miR-166ai    CM003604.1:10119875-10121023         2  Sbjct:      930 GGAAAAGAAGAGGAGAAT 913
                                                        Query:        1 CCTTTTTTTCACTTCTTA 18
                                                                        |||:|:|||| |||||||
Cc_miR-166ai    CM003604.1:22308739-22309861         2  Sbjct:      408 GGAGAGAAAGCGAAGAAT 391
```

```
                                                         Query:       1 CCTTTTTTTCACTTCTTA 18
                                                                        |||||||||| || ||||
Cc_miR-166ai     CM003605.1:15419859-15421840        2   Sbjct:    1454 GGAAAAAAAGAGACGAAT 1437

                                                         Query:       1 CCTTTTTTTCACTTCTTA 18
                                                                          ||||||||||||||||
Cc_miR-166ai     CM003606.1:12049408-12058727        2   Sbjct:    1804 ATAAAAAAAGTGAAGAAT 1787

                                                         Query:       1 CCTTTTTTTCACTTCTTA 18
                                                                        ||||||||||| || |||
Cc_miR-166ai     KQ483729.1:94363-100562             2   Sbjct:    5357 GGAAAAAAAGTTAAAAAT 5340

                                                         Query:       1 ATTGGTCCGGTTCAAT 16
                                                                        |:||||||| ||||||
Cc_miR-166d      CM003612.1:20907301-20909324      1.5   Sbjct:     517 TGACCAGGCGAAGTTA 502

                                                         Query:       1 ATTGGTCCGGTTCAAT 16
                                                                        |:||||||| ||||||
Cc_miR-166d      CM003612.1:20919404-20919905      1.5   Sbjct:     343 TGACCAGGCGAAGTTA 328

                                                         Query:       1 ATTTTCTCTCTACGTT 16
                                                                        |||||||||||||::|
Cc_miR-166f      KQ483455.1:404992-405704            1   Sbjct:     217 TAAAAGAGAGATGTGA 202

                                                         Query:       1 ATTTTCTCTCTACGTT 16
                                                                        |||||||| |||||||
Cc_miR-166f      KQ483570.1:155775-160347            1   Sbjct:     594 TAAAAGAGTGATGCAA 579

                                                         Query:       1 ATTTTCTCTCTACGTT 16
                                                                        ||||| |||||||:||
Cc_miR-166f      AGCT01040082.1:657-1375           1.5   Sbjct:     619 TAAAATAGAGATGTAA 604

                                                         Query:       1 ATTTTCTCTCTACGTT 16
                                                                         |||||||||:|||||
Cc_miR-166f      AGCT01046923.1:0-401              1.5   Sbjct:     390 AAAAAGAGAGGTGCAA 375

                                                         Query:       1 ATTTTCTCTCTACGTT 16
                                                                        |||||||||| ||||:
Cc_miR-166f      CM003603.1:15080991-15084547      1.5   Sbjct:    1784 TAAAAGAGAGTTGCAG 1769

                                                         Query:       1 ATTTTCTCTCTACGTT 16
                                                                         ||:||||||||||||
Cc_miR-166f      CM003606.1:7064892-7065505        1.5   Sbjct:     100 AAAGAGAGAGATGCAA 85
```

```
                                                      Query:        1 ATTTTCTCTCTACGTT 16
                                                                       ||||||||||||:||
Cc_miR-166f   KQ483670.1:175856-183475          1.5   Sbjct:     7584 AAAAAGAGAGATGTAA 7569

                                                      Query:        1 ATTTTCTCTCTACGTT 16
                                                                      ||||:||||||| |||
Cc_miR-166f   KQ483810.1:46190-47946            1.5   Sbjct:       81 TAAAGGAGAGATACAA 66

                                                      Query:        1 ATTTTCTCTCTACGTT 16
                                                                      ||||| |||||||:||
Cc_miR-166f   KQ484745.1:39350-42640            1.5   Sbjct:     1172 TAAAATAGAGATGTAA 1157

                                                      Query:        1 CAAAGGGATCGCATTGATCCTAAATCT 27
                                                                      ||||||||||||||||||||:|||||
Cc_miR-166g   KQ486010.1:2815-10076             1.5   Sbjct:     6809 GTTTCCCTAGCGTAACTAGGGTTTAGT 6783

                                                      Query:        1 CAAATTTTTCTAAACTTGTA 20
                                                                       |||:|:|||||||||||||
Cc_miR-166h   KQ483646.1:146077-149392            2   Sbjct:      775 CTTTGAGAAGATTTGAACAT 756

                                                      Query:        1 CAAATTTTTCTAAACTTGTA 20
                                                                       || ||||||||||||||||
Cc_miR-166h   KQ484096.1:107919-110361            2   Sbjct:     1646 TTTCAAAAAGATTTGAACAT 1627

                                                      Query:        1 CAAATTTTTCTAAACTTGTA 20
                                                                      ||||||| ||||| ||||||
Cc_miR-166h   KQ484311.1:46418-47340              2   Sbjct:      410 GTTTAAATAGATTGGAACAT 391

                                                      Query:        1 CACCTGCATGGCCAGA 16
                                                                      || |||||||||||:|
Cc_miR-166m   CM003604.1:5373991-5380514        1.5   Sbjct:     5186 GTTGACGTACCGGTTT 5171

                                                      Query:        1 CACCTGCATGGCCAGA 16
                                                                      ||  ||||||:|||||
Cc_miR-166m   KQ483497.1:370862-378485          2.5   Sbjct:     5126 GTATACGTACTGGTCT 5111

                                                      Query:        1 CAGCATCATCAAGATTCTCA 20
                                                                      ||||| ||||||||||| ||
Cc_miR-166n   CM003612.1:1832435-1832939          2   Sbjct:       59 GTCGTCGTAGTTCTAAGTGT 40

                                                      Query:        1 CAGGGCAATTCTCCTTTGGCAG 22
                                                                      | |||||| ||||||||||||:
Cc_miR-166q   CM003603.1:7699070-7707324        2.5   Sbjct:     6062 GGCCCGTTTAGAGGAAACCGTT 6041
```

```
                                                           Query:         1 CCAAAAGAAAAGCCAA 16
                                                                            |||||||||||||||
Cc_miR-166y    KQ483501.1:285901-287708              1     Sbjct:      1767 GGTTTTCTTTTCGGTG 1752
                                                           Query:         1 CCAAAAGAAAAGCCAA 16
                                                                            |||||||||||:|| |
Cc_miR-166y    CM003613.1:26233676-26235214        1.5     Sbjct:       427 GGTTTTCTTTTTGGAT 412
                                                           Query:         1 CCAAAAGAAAAGCCAA 16
                                                                            ||||||:|| |:||||
Cc_miR-166y    AGCT01028887.1:5-3659                 2     Sbjct:      2728 GGTTTTTTTATTGGTT 2713
                                                           Query:         1 CCAAAAGAAAAGCCAA 16
                                                                            ||||| || |||||||
Cc_miR-166y    CM003603.1:1209909-1212543            2     Sbjct:      1934 GGTTTGCTCTTCGGTT 1919
                                                           Query:         1 CCAAAAGAAAAGCCAA 16
                                                                            |||||||||||  |||
Cc_miR-166y    CM003604.1:22687989-22689796          2     Sbjct:       824 GGTTTTCTTTTACGTT 809
                                                           Query:         1 CCAAAAGAAAAGCCAA 16
                                                                            ||| |||| |||||||
Cc_miR-166y    CM003606.1:1885637-1886734            2     Sbjct:       613 GGTCTTCTGTTCGGTT 598
                                                           Query:         1 CCAAAAGAAAAGCCAA 16
                                                                            ||||||:||||:|||
Cc_miR-166y    CM003606.1:4278738-4291510            2     Sbjct:      3564 GGTTTTTTTTTTGGTG 3549
                                                           Query:         1 CCAAAAGAAAAGCCAA 16
                                                                            ||||||| | ||||||
Cc_miR-166y    CM003608.1:7374129-7378753            2     Sbjct:      4601 GGTTTTCGTATCGGTT 4586
                                                           Query:         1 CCAAAAGAAAAGCCAA 16
                                                                             |||||:||||:||||
Cc_miR-166y    CM003609.1:9585470-9589026            2     Sbjct:       445 CGTTTTTTTTTTGGTT 430
                                                           Query:         1 CCAAAAGAAAAGCCAA 16
                                                                            | ||||||||| ||||
Cc_miR-166y    CM003610.1:9206589-9207393            2     Sbjct:       228 GTTTTTCTTTTGGGTT 213
                                                           Query:         1 CCAAAAGAAAAGCCAA 16
                                                                             |||||||||||| ||
Cc_miR-166y    CM003611.1:6648011-6649520            2     Sbjct:        95 AGTTTTCTTTTCGTTT 80
```

```
                                                           Query:         1 CCAAAAGAAAAGCCAA 16
                                                                            ||||||:||||: |||
Cc_miR-166y       KQ483558.1:178490-181243              2  Sbjct:       753 GGTTTTTTTTTTTGTT 738
                                                           Query:         1 CCAAAAGAAAAGCCAA 16
                                                                            ||||||:||||:| ||
Cc_miR-166y       KQ483714.1:78637-79486                2  Sbjct:       434 GGTTTTTTTTTTGTTT 419
                                                           Query:         1 CCAAAAGAAAAGCCAA 16
                                                                            ||||||||| |:|||
Cc_miR-166y       AGCT01060069.1:3703-4058            2.5  Sbjct:        45 GGTTTTCTTCTTGGTC 30
                                                           Query:         1 CCACATTTTTCTTAAAC 17
                                                                             |||||||||||||||
Cc_miR-166z       CM003606.1:12282840-12286404          2  Sbjct:      2422 AGTGTAAAAAGAATTTT 2406
                                                           Query:         1 CCACATTTTTCTTAAAC 17
                                                                            |||||| |:|||:||||
Cc_miR-166z       CM003608.1:3412187-3420951            2  Sbjct:       895 GGTGTATAGAGAGTTTG 879
                                                           Query:         1 CCACATTTTTCTTAAAC 17
                                                                            ||| ||||||||||| |
Cc_miR-166z       CM003612.1:427796-431350              2  Sbjct:      1158 GGTATAAAAAGAATTAG 1142
                                                           Query:         1 CTACACCTCTCAAGTC 16
                                                                            ||||| |||| |||||
Cc_miR-167c       CM003604.1:15225003-15228624          2  Sbjct:      2608 GATGTAGAGAATTCAG 2593
                                                           Query:         1 CTACACCTCTCAAGTC 16
                                                                            |:|||||||||||: |
Cc_miR-167c       KQ483612.1:196102-203262              2  Sbjct:       542 GGTGTGGAGAGTTTTG 527
                                                           Query:         1 CTCCCTGAATGCCAT 15
                                                                            ||| ||||||| |||
Cc_miR-167f       CM003605.1:23031873-23037221          2  Sbjct:       516 GAGAGACTTACAGTA 502
                                                           Query:         1 CTCCCTGAATGCCAT 15
                                                                            |||||| ||||| ||
Cc_miR-167f       CM003608.1:10699986-10706027          2  Sbjct:      1146 GAGGGAATTACGATA 1132
                                                           Query:         1 CTCCCTGAATGCCAT 15
                                                                            | || ||||||||||
Cc_miR-167f       CM003611.1:4408918-4412809            2  Sbjct:      3142 GTGGAACTTACGGTA 3128
```

```
                                                  Query:     1 CTCCCTGAATGCCAT 15
                                                                |||||||||| |||
Cc_miR-167f   CM003612.1:675195-676653         2  Sbjct:   266 CAGGGACTTACAGTA 252
                                                  Query:     1 CTCCCTGAATGCCAT 15
                                                                ||| ||||||||||
Cc_miR-167f   CM003613.1:41592718-41597796     2  Sbjct:  2608 AAGGTACTTACGGTA 2594
                                                  Query:     1 CTCCCTGAATGCCAT 15
                                                               | || ||||||||||
Cc_miR-167f   KQ483656.1:65948-66468           2  Sbjct:    20 GTGGAACTTACGGTA 6
                                                  Query:     1 CTTTCTTGAACTGTGTA 17
                                                               ||||||||||||||:|
Cc_miR-167m   KQ483722.1:93247-94448         1.5  Sbjct:   926 GAAAGAACTTGACATAG 910
                                                  Query:     1 CTTTCTTGAACTGTGTA 17
                                                               |||:|:|| ||||||||
Cc_miR-167m   KQ483437.1:546005-548047         2  Sbjct:  1516 GAAGGGACGTGACACAT 1500
                                                  Query:     1 CTTTCTTGAACTGTGTA 17
                                                               ||:||||||| |||:||
Cc_miR-167m   KQ484806.1:12609-19288           2  Sbjct:  4757 GAGAGAACTTAACATAT 4741
                                                  Query:     1 GAATTTAAACTTCAG 15
                                                               ||||||||| |||||
Cc_miR-167s   CM003603.1:8823571-8827055       1  Sbjct:  1422 CTTAAATTTTAAGTC 1408
                                                  Query:     1 GAATTTAAACTTCAG 15
                                                               :||||||||||| ||
Cc_miR-167s   AGCT01045941.1:697-4729        1.5  Sbjct:   765 TTTAAATTTGAAATC 751
                                                  Query:     1 GAATTTAAACTTCAG 15
                                                               :|||:|||||||||:
Cc_miR-167s   AGCT01059850.1:0-239           1.5  Sbjct:    97 TTTAGATTTGAAGTT 83
                                                  Query:     1 GAATTTAAACTTCAG 15
                                                               :||||||||||| ||
Cc_miR-167s   AGCT01067810.1:500-1817        1.5  Sbjct:   338 TTTAAATTTGAAATC 324
                                                  Query:     1 GAATTTAAACTTCAG 15
                                                               |||||:||||||||
Cc_miR-167s   CM003609.1:5829715-5831921     1.5  Sbjct:  1832 CTTAAGTTTGAAGTA 1818
```

```
                                                         Query:          1 GAATTTAAACTTCAG 15
                                                                           :||||||||||||||
Cc_miR-167s      CM003611.1:4192668-4202075          1.5 Sbjct:       6837 TTTAAATTTGAAGTA 6823
                                                         Query:          1 GAATTTAAACTTCAG 15
                                                                           ||| ||||||||||:
Cc_miR-167s      KQ483412.1:1764462-1765218          1.5 Sbjct:        613 CTTTAATTTGAAGTT 599
                                                         Query:          1 GAATTTAAACTTCAG 15
                                                                           :|||||||| |||||
Cc_miR-167s      KQ483440.1:534010-540949            1.5 Sbjct:       2699 TTTAAATTTTAAGTC 2685
                                                         Query:          1 GAATTTAAACTTCAG 15
                                                                           |||||||||||:|||
Cc_miR-167s      KQ483564.1:175621-177126            1.5 Sbjct:        474 CTTAAATTTGGAGTA 460
                                                         Query:          1 GAGCTCCCTTCACGTCAA 18
                                                                           ||| |||||||| :||||
Cc_miR-168e      CM003603.1:15157275-15170539        2.5 Sbjct:       3276 CTCCAGGGAAGTATAGTT 3259
                                                         Query:          1 GAGCTCCCTTCACGTCAA 18
                                                                           ||||||||||||  :|||
Cc_miR-168e      CM003608.1:4341208-4343428          2.5 Sbjct:        866 CTCGAGGGAAGTTAGGTT 849
                                                         Query:          1 GAGGGGAATGAAGCCTG 17
                                                                           |||||||| |||: |||
Cc_miR-168g      KQ484158.1:14911-15235              2.5 Sbjct:        195 CTCCCCTTCCTTTAGAC 179
                                                         Query:          1 GATAAATTTTTCTAGA 16
                                                                           :|||||||||||||||
Cc_miR-168i      CM003612.1:16431584-16433003        0.5 Sbjct:        971 TTATTTAAAAAGATCT 956
                                                         Query:          1 GATAAATTTTTCTAGA 16
                                                                           ||||||:|||||||||
Cc_miR-168i      KQ483485.1:242489-245051            0.5 Sbjct:       1395 CTATTTGAAAAGATCT 1380
                                                         Query:          1 GATAAATTTTTCTAGA 16
                                                                           ||||||:||:||||||
Cc_miR-168i      CM003606.1:7048767-7052030            1 Sbjct:       2351 CTATTTGAAGAGATCT 2336
                                                         Query:          1 GATAAATTTTTCTAGA 16
                                                                           ||||||:||:||||||
Cc_miR-168i      KQ483558.1:178490-181243              1 Sbjct:       1343 CTATTTGAAGAGATCT 1328
```

```
                                                               Query:        1 GATAAATTTTTCTAGA 16
                                                                                |||||||||||||||
Cc_miR-168i    KQ484070.1:90162-95418                   1      Sbjct:     4689 ATATTTAAAAAGATCT 4674
                                                               Query:        1 GATAAATTTTTCTAGA 16
                                                                               ||||||||| ||||:|
Cc_miR-168i    AGCT01065792.1:11-793                  1.5      Sbjct:      622 CTATTTAAATAGATTT 607
                                                               Query:        1 GATAAATTTTTCTAGA 16
                                                                               |||||||||:|||| |
Cc_miR-168i    CM003603.1:12295118-12299992           1.5      Sbjct:     3234 CTATTTAAAGAGATAT 3219
                                                               Query:        1 GATAAATTTTTCTAGA 16
                                                                               || |||||||:|||||
Cc_miR-168i    CM003606.1:9959158-9960010             1.5      Sbjct:      731 CTTTTTAAAAGGATCT 716
                                                               Query:        1 GATAAATTTTTCTAGA 16
                                                                               :||||||||:||:|||
Cc_miR-168i    CM003608.1:9254192-9258501             1.5      Sbjct:     3020 TTATTTAAAGAGGTCT 3005
                                                               Query:        1 GATAAATTTTTCTAGA 16
                                                                               |||||||||:||:|:|
Cc_miR-168i    CM003609.1:2794127-2798852             1.5      Sbjct:     2316 CTATTTAAAGAGGTTT 2301
                                                               Query:        1 GATAAATTTTTCTAGA 16
                                                                               :||||||||:||:|||
Cc_miR-168i    CM003609.1:3506223-3510535             1.5      Sbjct:     3497 TTATTTAAAGAGGTCT 3482
                                                               Query:        1 GATAAATTTTTCTAGA 16
                                                                               |||||| ||:||||||
Cc_miR-168i    CM003611.1:168691-172547               1.5      Sbjct:     1041 CTATTTTAAGAGATCT 1026
                                                               Query:        1 GATAAATTTTTCTAGA 16
                                                                               :|||||||| ||||||
Cc_miR-168i    CM003613.1:36729568-36731568           1.5      Sbjct:      692 TTATTTAAATAGATCT 677
                                                               Query:        1 GATAAATTTTTCTAGA 16
                                                                               |||||||||:|| |||
Cc_miR-168i    KQ483429.1:578911-579246               1.5      Sbjct:      299 CTATTTAAAGAGTTCT 284
                                                               Query:        1 GATAAATTTTTCTAGA 16
                                                                               |||||||||:||:|:|
Cc_miR-168i    KQ483600.1:274294-279137               1.5      Sbjct:      863 CTATTTAAAGAGGTTT 848
```

```
                                                Query:      1 GATAAATTTTTCTAGA 16
                                                              ||||||| ||||:|||
Cc_miR-168i   KQ483918.1:56272-57636       1.5  Sbjct:    319 CTATTTATAAAGGTCT 304
                                                Query:      1 GATAAATTTTTCTAGA 16
                                                              ||||||:||||||| |
Cc_miR-168i   KQ484094.1:79807-85492       1.5  Sbjct:   4316 CTATTTGAAAAGATAT 4301
                                                Query:      1 GATAAATTTTTCTAGA 16
                                                              :|||||||||| ||||
Cc_miR-168i   KQ486010.1:2815-10076        1.5  Sbjct:   3359 TTATTTAAAAAAATCT 3344
                                                Query:      1 GATAAATTTTTCTAGA 16
                                                              ||:||||| |||||:|
Cc_miR-168i   AGCT01016574.1:466-943         2  Sbjct:    417 CTGTTTAATAAGATTT 402
                                                Query:      1 GATATTGGTACGGTTCAAT 19
                                                              ||||||||:||||:|||||
Cc_miR-168j   KQ483652.1:191566-195382       1  Sbjct:    291 CTATAACCGTGCCGAGTTA 273
                                                Query:      1 GATGAATGAAGAC 13
                                                              |||||||:|||||
Cc_miR-168l   CM003613.1:29244730-29246922 0.5  Sbjct:    424 CTACTTATTTCTG 412
                                                Query:      1 GATGTAGCATCATCAAG 17
                                                              ||||||:||||| |||
Cc_miR-169a   CM003604.1:16965825-16970692 2.5  Sbjct:    679 CTACATTGTAGTTGTTA 663
                                                Query:      1 GATGTAGCATCATCAAG 17
                                                              :||:||||||||:|||
Cc_miR-169a   CM003604.1:4033871-4038538   2.5  Sbjct:   1645 TTATATCGTAGTGGTTA 1629
                                                Query:      1 GATGTAGCATCATCAAG 17
                                                              ||||:||||| || |||
Cc_miR-169a   CM003608.1:212234-215046     2.5  Sbjct:    225 CTACGTCGTACTAATTC 209
                                                Query:      1 GATGTAGCATCATCAAG 17
                                                              || |||:||| ||||||
Cc_miR-169a   CM003609.1:5653711-5662230   2.5  Sbjct:   2322 CTTCATTGTACTAGTTC 2306
                                                Query:      1 GATGTAGCATCATCAAG 17
                                                              | ||:|||| |||||||
Cc_miR-169a   CM003612.1:1832435-1832939   2.5  Sbjct:     63 CGACGTCGTCGTAGTTC 47
```

```
                                                  Query:      1 GATGTAGCATCATCAAG 17
                                                                 |:|||| |||||||||
Cc_miR-169a    KQ483525.1:407396-409104     2.5   Sbjct:   1454 ATGCATCTTAGTAGTTC 1438

                                                  Query:      1 GATGTAGCATCATCAAGATTCA 22
                                                                | ||:|||| ||||||||||||
Cc_miR-169b    CM003612.1:1832435-1832939   2.5   Sbjct:     63 CGACGTCGTCGTAGTTCTAAGT 42

                                                  Query:      1 GCATGAAGTTTGGTTAT 17
                                                                |||||||||||| |||:
Cc_miR-171f    AGCT01020542.1:215-1275      1.5   Sbjct:    900 CGTACTTCAAACAAATG 884

                                                  Query:      1 GCATGAAGTTTGGTTAT 17
                                                                |||||||| ||::||||
Cc_miR-171f    KQ483452.1:392755-393252       2   Sbjct:     93 CGTACTTCCAATTAATA 77

                                                  Query:      1 GCATGAAGTTTGGTTAT 17
                                                                :|||:||:|||:|||||
Cc_miR-171f    KQ483565.1:350961-365428       2   Sbjct:   2798 TGTATTTTAAATCAATA 2782

                                                  Query:      1 GCATGAAGTTTGGTTAT 17
                                                                :||||||::|||||:||
Cc_miR-171f    KQ483730.1:73063-78914         2   Sbjct:   3382 TGTACTTTGAACCAGTA 3366

                                                  Query:      1 GCGACTTGTATTTTAGCCCT 20
                                                                | ||||||| |||||:||||
Cc_miR-171h    CM003612.1:427796-431350     2.5   Sbjct:   1826 CACTGAACAAAAAATTGGGA 1807

                                                  Query:      1 GCTTAATCCGCTATTCTAAGATGGTAT 27
                                                                |||||||||:||||||||| |||||||
Cc_miR-171i    CM003612.1:20929167-20941402 1.5   Sbjct:   5822 CGAATTAGGTGATAAGATTGTACCATA 5796

                                                  Query:      1 GGAACAGGCAGAGCAT 16
                                                                :||||||| |:|||||
Cc_miR-171k    CM003605.1:12328333-12332742   2   Sbjct:    158 TCTTGTCCTTTTCGTA 143

                                                  Query:      1 GGAACAGGCAGAGCAT 16
                                                                |:|| |:|||||||||
Cc_miR-171k    KQ483574.1:323149-323467       2   Sbjct:    248 CTTTTTTCGTCTCGTA 233

                                                  Query:      1 GGAACAGGCAGAGCAT 16
                                                                |||| |||||| ||||
Cc_miR-171k    KQ484839.1:38368-39562         2   Sbjct:    962 CCTTCTCCGTCACGTA 947
```

```
                                                                   Query:         1 GGAGCTCCCTTCAGTCCA 18
                                                                                    :||||||||||||:||||
Cc_miR-171m     CM003608.1:4341208-4343428                   1     Sbjct:       867 TCTCGAGGGAAGTTAGGT 850

                                                                   Query:         1 TAATTGCCTAATCTCTAGTCGATGTG 26
                                                                                    :|||||||||||||||||||||||||
Cc_miR-172aa    KQ483469.1:394906-406708                   0.5     Sbjct:      4574 GTTAACGGATTAGAGATCAGCTACAC 4549

                                                                   Query:         1 TAATTGCCTAATCTCTAGTCGATGTG 26
                                                                                    :|||||||||||||||||||||||||
Cc_miR-172aa    KQ483469.1:394906-406708                   0.5     Sbjct:      4216 GTTAACGGATTAGAGATCAGCTACAC 4191

                                                                   Query:         1 TAATTGCCTAATCTCTAGTCGATGTG 26
                                                                                    :|||||||||||||||||||||||||
Cc_miR-172aa    KQ486010.1:2815-10076                      0.5     Sbjct:      2897 GTTAACGGATTAGAGATCAGCTACAC 2872

                                                                   Query:         1 TAATTGCCTAATCTCTAGTCGATGTG 26
                                                                                    :||||||||||||||||||||||::|
Cc_miR-172aa    KQ486010.1:2815-10076                      1.5     Sbjct:      3207 GTTAACGGATTAGAGATCAGCTATGC 3182

                                                                   Query:         1 TACAAATTAGCTTATACATAAACTAAT 27
                                                                                    |||||||||||||  ||||||||||||
Cc_miR-172ab    KQ484452.1:33191-36713                       2     Sbjct:      2783 ATGTTTAATCGAACCTGTATTTGATTA 2757

                                                                   Query:         1 TACAAATTAGCTTATACATAAACTAAT 27
                                                                                    |||||||||: ||||||||||| ||||
Cc_miR-172ab    KQ483474.1:457756-458495                   2.5     Sbjct:       327 ATGTTTAATTAAATATGTATTTAATTA 301

                                                                   Query:         1 TATACTCATTTCATAA 16
                                                                                    |||||:|||||||| |
Cc_miR-172ad    CM003609.1:14350001-14360764               1.5     Sbjct:      5439 ATATGGGTAAAGTAAT 5424

                                                                   Query:         1 TATACTCATTTCATAA 16
                                                                                    |||||:| ||||||||
Cc_miR-172ad    CM003612.1:7569934-7572859                 1.5     Sbjct:      2007 ATATGGGGAAAGTATT 1992

                                                                   Query:         1 TATATTGAAAAAAATCCAAGTCCC 24
                                                                                    | ||||| ||||||:|||||||||
Cc_miR-172ae    CM003611.1:4192668-4202075                 2.5     Sbjct:      5161 ACATAACCTTTTTTGGGTTCAGGG 5138

                                                                   Query:         1 TATTCAAACATGTATATAA 19
                                                                                    |||||| ||||:|||||||
Cc_miR-172ag    CM003604.1:10935798-10937675               1.5     Sbjct:       837 ATAAGTATGTATATATATT 819
```

```
                                                          Query:         1 TATTCAAACATGTATATAA 19
                                                                           ||:||||||| |||:||||
Cc_miR-172ag    CM003604.1:33771977-33773894          2   Sbjct:       945 ATGAGTTTGTTCATGTATT 927

                                                          Query:         1 GGAGTGAGGCTGTCACAG 18
                                                                           |:|||||:|||:||| ||
Cc_miR-172b     KQ483411.1:1848576-1849580          2.5   Sbjct:        65 CTTCACTTCGATAGTATC 48

                                                          Query:         1 GGATTTAAGCCTAACT 16
                                                                           |||||||||||||| |
Cc_miR-172e     KQ483719.1:248121-255165              1   Sbjct:      6623 CCTAAATTCGGATTAA 6608

                                                          Query:         1 GGATTTAAGCCTAACT 16
                                                                           |:|||||||| |||||
Cc_miR-172e     CM003609.1:14844280-14847551        1.5   Sbjct:      2493 CTTAAATTCGAATTGA 2478

                                                          Query:         1 GGATTTAAGCCTAACT 16
                                                                           :| ||:||||||||||
Cc_miR-172e     CM003608.1:20651564-20652882          2   Sbjct:       982 TCAAAGTTCGGATTGA 967

                                                          Query:         1 GGATTTAAGCCTAACT 16
                                                                           ::||||||| ||||||
Cc_miR-172e     KQ483629.1:273349-278921              2   Sbjct:      2729 TTTAAATTCAGATTGA 2714

                                                          Query:         1 GGTGACAGAAGAGAGTGAGCACACATGGC 29
                                                                           ||||||||| |||||||||||||||||||
Cc_miR-172j     CM003612.1:3998296-3999928            2   Sbjct:      1434 CCACTGTCTGCTCTCACTCGTGTGTACCA 1406

                                                          Query:         1 GTGAGAATGAGAGGAGGGAGAG 22
                                                                           ||:|:|||:|||||||:||||:
Cc_miR-172l     KQ484260.1:6421-9436                2.5   Sbjct:      1505 CATTTTTATTCTCCTCTCTCTT 1484

                                                          Query:         1 GTAGCGGTTCATCGAT 16
                                                                           |||:| ||||||| ||
Cc_miR-172p     CM003613.1:38322431-38325206        2.5   Sbjct:      1756 CATTGACAAGTAGGTA 1741

                                                          Query:         1 GTAGCGGTTCATCGAT 16
                                                                           |:|: ||:||||||||
Cc_miR-172p     KQ483427.1:158733-161174            2.5   Sbjct:       358 CGTTACCGAGTAGCTA 343

                                                          Query:         1 GTAGCGGTTCATCGAT 16
                                                                           | |:|:|||||||:||
Cc_miR-172p     KQ485607.1:22879-24940              2.5   Sbjct:      1287 CTTTGTCAAGTAGTTA 1272
```

```
                                                        Query:          1 GTATCTATGTATGTAA 16
                                                                          |||| ||||:||||||
Cc_miR-172q      CM003603.1:12295118-12299992      1.5  Sbjct:       1530 CATAAATACGTACATT 1515

                                                        Query:          1 GTATCTATGTATGTAA 16
                                                                          :||||:||:|||||||
Cc_miR-172q      CM003603.1:9311152-9312466        1.5  Sbjct:        186 TATAGGTATATACATT 171

                                                        Query:          1 GTATCTATGTATGTAA 16
                                                                          :||:|||||||:||||
Cc_miR-172q      CM003613.1:14251860-14255138      1.5  Sbjct:       2244 TATGGATACATGCATT 2229

                                                        Query:          1 GTATCTATGTATGTAA 16
                                                                          :|||||||:||||:||
Cc_miR-172q      KQ483612.1:196102-203262          1.5  Sbjct:        179 TATAGATATATACGTT 164

                                                        Query:          1 GTATCTATGTATGTAA 16
                                                                           :||||||||||||||
Cc_miR-172q      KQ483660.1:225666-232864          1.5  Sbjct:       1766 AGTAGATACATACATT 1751

                                                        Query:          1 GTCCTGCTTCATCAAGTG 18
                                                                          ||||||||:||:|||:|:
Cc_miR-172t      CM003610.1:12106784-12109330        2  Sbjct:       1996 CAGGACGAGGTGGTTTAT 1979

                                                        Query:          1 GTCCTGCTTCATCAAGTG 18
                                                                          :|||||||:||:| ||||
Cc_miR-172t      CM003604.1:34856603-34858180      2.5  Sbjct:        848 TAGGACGAGGTGGGTCAC 831

                                                        Query:          1 GTCCTGCTTCATCAAGTG 18
                                                                          |||| :|||| |||||||
Cc_miR-172t      CM003613.1:30617103-30618040      2.5  Sbjct:        744 CAGGTTGAAGAAGTTCAC 727

                                                        Query:          1 GTGATTATTATAATAGC 17
                                                                          ||||||||:|||||| |
Cc_miR-172u      CM003613.1:43385546-43388896      1.5  Sbjct:        798 CACTAATAGTATTATAG 782

                                                        Query:          1 GTGTGGAAGAATCTGTAAAGC 21
                                                                          |||||:||:|||||||||||:
Cc_miR-172v      AGCT01055338.1:0-1528             2.5  Sbjct:       1214 CACACTTTTTTAGACATTTTA 1194

                                                        Query:          1 GTGTGGAAGAATCTGTAAAGC 21
                                                                          : ||:|||||||||:||||||
Cc_miR-172v      CM003609.1:620372-621530          2.5  Sbjct:        450 TTCATCTTCTTAGATATTTCG 430
```

```
                                                        Query:      1 GTGTTTGTGTGGAGGGTGAAA 21
                                                                      | |||||||||:| |||||||
Cc_miR-172w    CM003605.1:928242-930877         2.5     Sbjct:   1077 CCCAAACACACTTACCACTTT 1057

                                                        Query:      1 TAGCTGGTTCCCTCCGAA 18
                                                                      ||| |||:|||||||:||
Cc_miR-319aa   KQ483973.1:52973-61100             2     Sbjct:   6767 ATCAACCGAGGGAGGTTT 6750

                                                        Query:      1 TATGGAATGAGAGAGACAA 19
                                                                      |||::|||:|:||||||||
Cc_miR-319ai   CM003609.1:2462642-2464744         2     Sbjct:   1658 ATATTTTATTTTCTCTGTT 1640

                                                        Query:      1 TATGGAATGAGAGAGACAA 19
                                                                      |||:||||||||:|:| ||
Cc_miR-319ai   CM003612.1:20398860-20400211     2.5     Sbjct:    799 ATATCTTACTCTTTTTCTT 781

                                                        Query:      1 TATGGAATGAGAGAGACAA 19
                                                                       |||||||||:|:|:||||
Cc_miR-319ai   KQ483846.1:124118-126218         2.5     Sbjct:    198 CTACCTTACTTTTTTTGTT 180

                                                        Query:      1 TATGGAATGAGAGAGACAA 19
                                                                      :||: |||||:||||||||
Cc_miR-319ai   KQ484274.1:11274-13571           2.5     Sbjct:   1768 GTATATTACTTTCTCTGTT 1750

                                                        Query:      1 TATGGAATGAGAGAGACAA 19
                                                                      |||::|||:|:||||||||
Cc_miR-319aj   CM003609.1:2462642-2464744         2     Sbjct:   1658 ATATTTTATTTTCTCTGTT 1640

                                                        Query:      1 TATGGAATGAGAGAGACAA 19
                                                                      |||:||||||||:|:| ||
Cc_miR-319aj   CM003612.1:20398860-20400211     2.5     Sbjct:    799 ATATCTTACTCTTTTTCTT 781

                                                        Query:      1 TATGGAATGAGAGAGACAA 19
                                                                       |||||||||:|:|:||||
Cc_miR-319aj   KQ483846.1:124118-126218         2.5     Sbjct:    198 CTACCTTACTTTTTTTGTT 180

                                                        Query:      1 TATGGAATGAGAGAGACAA 19
                                                                      :||: |||||:||||||||
Cc_miR-319aj   KQ484274.1:11274-13571           2.5     Sbjct:   1768 GTATATTACTTTCTCTGTT 1750

                                                        Query:      1 TATTGTCTTCCTTCGTCT 18
                                                                      ||:::|||:|||||:|||
Cc_miR-319am   KQ484558.1:55752-56638           2.5     Sbjct:    805 ATGGTAGAGGGAAGTAGA 788
```

```
                                                               Query:          1 TATTGTCTTCCTTCGTCT 18
                                                                                 |||||| || ||||:|||
Cc_miR-319am     KQ485437.1:23558-26967                   2.5  Sbjct:        722 ATAACAAAATGAAGTAGA 705

                                                               Query:          1 TGAATTTAAACTTCAG 16
                                                                                 |||||||||| |||||
Cc_miR-319c      CM003603.1:8823571-8827055                 1  Sbjct:       1423 ACTTAAATTTTAAGTC 1408

                                                               Query:          1 TGAATTTAAACTTCAG 16
                                                                                 |:||||||||||| ||
Cc_miR-319c      AGCT01045941.1:697-4729                  1.5  Sbjct:        766 ATTTAAATTTGAAATC 751

                                                               Query:          1 TGAATTTAAACTTCAG 16
                                                                                 |:||||||||||| ||
Cc_miR-319c      AGCT01067810.1:500-1817                  1.5  Sbjct:        339 ATTTAAATTTGAAATC 324

                                                               Query:          1 TGAATTTAAACTTCAG 16
                                                                                 ||||||||||||:|||
Cc_miR-319c      KQ483564.1:175621-177126                 1.5  Sbjct:        475 ACTTAAATTTGGAGTA 460

                                                               Query:          1 TTCCTTTTTTTCACTTCTTA 20
                                                                                 ||||||||:||| |:|||||
Cc_miR-319i      CM003604.1:10119875-10121023               2  Sbjct:        932 AAGGAAAAGAAGAGGAGAAT 913

                                                               Query:          1 TTCCTTTTTTTCACTTCTTA 20
                                                                                 :|  ||||||||||||||||
Cc_miR-319i      CM003606.1:12049408-12058727             2.5  Sbjct:       1806 GAATAAAAAAAGTGAAGAAT 1787

                                                               Query:          1 TTCCTTTTTTTCACTTCTTA 20
                                                                                 |||||||||||| ||||:|
Cc_miR-319i      KQ483638.1:228486-237554                 2.5  Sbjct:       8292 AAGGAAAAAAAGAGAAGGAA 8273

                                                               Query:          1 TTCCTTTTTTTCACTTCTTA 20
                                                                                 :|||||||||||| || |||
Cc_miR-319i      KQ483729.1:94363-100562                  2.5  Sbjct:       5359 GAGGAAAAAAAGTTAAAAAT 5340

                                                               Query:          1 TTCTAGAAGAAAAAAGTATGAAGGAAAAC 29
                                                                                 |||||||||||||||:|||||| |||||
Cc_miR-319j      CM003604.1:17771749-17777173             2.5  Sbjct:       1221 AAGATCTTCTTTTTTTATACTTACTTTTT 1193

                                                               Query:          1 TTTCCTTTTTTTTCACTTCTTAT 22
                                                                                 |:|  |||||||||||||||||
Cc_miR-319l      CM003606.1:12049408-12058727             2.5  Sbjct:       1807 AGAATAAAAAAAAGTGAAGAATA 1786
```

```
                                                         Query:        1 TAAGCAATAGGGCACC 16
                                                                         ||||| |||||:|| |
Cc_miR-319o   CM003604.1:14355819-14360536         2.5  Sbjct:     4016 ATTCGATATCCTGTTG 4001
                                                         Query:        1 TAAGCAATAGGGCACC 16
                                                                         :|| ||| ||||||||
Cc_miR-319o   CM003604.1:16021980-16023736         2.5  Sbjct:     1104 GTTGGTTTTCCCGTGG 1089
                                                         Query:        1 TAAGCAATAGGGCACC 16
                                                                         :||| ||:|:||||||
Cc_miR-319o   CM003613.1:20167735-20170274         2.5  Sbjct:     2346 GTTCCTTGTTCCGTGG 2331
                                                         Query:        1 TAAGCAATAGGGCACC 16
                                                                         :||:|||:|:|:||||
Cc_miR-319o   CM003613.1:47276639-47278710         2.5  Sbjct:     1919 GTTTGTTGTTCTGTGG 1904
                                                         Query:        1 TAAGCAATAGGGCACC 16
                                                                         ||||||||| : ||||
Cc_miR-319o   KQ483558.1:78637-82195               2.5  Sbjct:     2070 ATTCGTTATATAGTGG 2055
                                                         Query:        1 TAAGCAATAGGGCACC 16
                                                                         | |:||||||::||||
Cc_miR-319o   KQ483789.1:110254-114569             2.5  Sbjct:     2872 AATTGTTATCTTGTGG 2857
                                                         Query:        1 TAGAGAGTGAGCACACAT 18
                                                                         |||||||||||||| |||
Cc_miR-319u   KQ483994.1:22970-25497                 1  Sbjct:      469 ATCTCTCACTCGTGCGTA 452
                                                         Query:        1 TAGAGAGTGAGCACACAT 18
                                                                           ||||||||||||||||
Cc_miR-319u   CM003612.1:3998296-3999928             2  Sbjct:     1426 TGCTCTCACTCGTGTGTA 1409
                                                         Query:        1 TAGAGAGTGAGCACACAT 18
                                                                         ||||||:|||| | ||||
Cc_miR-319u   AGCT01021878.1:6804-8451             2.5  Sbjct:     1528 ATCTCTTACTCTTATGTA 1511
                                                         Query:        1 TCAAACCAACATGCTTGT 18
                                                                         || |||||| ||||||||
Cc_miR-390c   CM003611.1:9002648-9003068             2  Sbjct:       35 AGGTTGGTTTTACGAACA 18
                                                         Query:        1 TCAAACCAACATGCTTGT 18
                                                                         ||||| |||||| |||:|
Cc_miR-390c   CM003604.1:15021532-15028098         2.5  Sbjct:     2118 AGTTTCGTTGTAAGAATA 2101
```

```
                                                                   Query:      1 TCAAACCAACATGCTTGT 18
                                                                                 ||||| |||||:||| ||
Cc_miR-390c      CM003604.1:31762329-31763487        2.5           Sbjct:    166 AGTTTAGTTGTGCGATCA 149

                                                                   Query:      1 TCAGTTGGAGTGCTTTTA 18
                                                                                 | |||||||:||||||||
Cc_miR-390i      KQ484825.1:1639-2869                1.5           Sbjct:   1119 AATCAACCTTACGAAAAT 1102

                                                                   Query:      1 TCAGTTGGAGTGCTTTTA 18
                                                                                 ||| :|:||||:||||||
Cc_miR-390i      CM003612.1:5550117-5555516          2.5           Sbjct:   4213 AGTAGATCTCATGAAAAT 4196

                                                                   Query:      1 TCAGTTGGAGTGCTTTTA 18
                                                                                 |||:|: |||||||:|||
Cc_miR-390i      KQ483425.1:294512-295382            2.5           Sbjct:    514 AGTTAGGCTCACGAGAAT 497

                                                                   Query:      1 TCAGTTGGAGTGCTTTTA 18
                                                                                 |||:||:|| |:||||||
Cc_miR-390i      KQ483716.1:181828-182233            2.5           Sbjct:    347 AGTTAATCTAATGAAAAT 330

                                                                   Query:      1 TCAGTTGGAGTGCTTTTA 18
                                                                                 | |||||:||| ||||||
Cc_miR-390i      KQ483828.1:98544-101155             2.5           Sbjct:    671 ATTCAACTTCAAGAAAAT 654

                                                                   Query:      1 TCCAAAGGGATCGCATTGATCCTAAATCT 29
                                                                                 ||||||||||||||||||||||:||||||
Cc_miR-390m      KQ486010.1:2815-10076               1.5           Sbjct:   6811 AGGTTTCCCTAGCGTAACTAGGGTTTAGT 6783

                                                                   Query:      1 TCCACAGCTTTCTTGAACTG 20
                                                                                 ||||||||| ||||||| ||
Cc_miR-390n      CM003605.1:10604013-10604904          2           Sbjct:    164 AGGTGTCGATAGAACTTAAC 145

                                                                   Query:      1 TCGGAGAGAGACTCGGTG 18
                                                                                 ||||||||||| || :||
Cc_miR-393d      CM003612.1:4061894-4063642          2.5           Sbjct:   1631 AGCCTCTCTCTCAGGTAC 1614

                                                                   Query:      1 TCTTATATTGAAATCAAC 18
                                                                                 ||||||||| |||| |||
Cc_miR-393j      CM003607.1:4977317-4978874            2           Sbjct:    596 AGAATATAAGTTTATTTG 579

                                                                   Query:      1 TCTTATATTGAAATCAAC 18
                                                                                 | |||||||||||| |||
Cc_miR-393j      CM003612.1:13342321-13346053          2           Sbjct:   2869 AAAATATAACTTTACTTG 2852
```

```
                                                   Query:       1 TCTTATATTGAAATCAAC 18
                                                                  |||||||:| |||||||
Cc_miR-393j   CM003612.1:5550117-5555516      2.5  Sbjct:    2772 AGAATATGAATTTAGTTA 2755
                                                   Query:       1 TCTTATATTGAAATCAAC 18
                                                                   ||:|| |||||||||||
Cc_miR-393j   KQ483638.1:228486-237554        2.5  Sbjct:    1845 TGAGTAAAACTTTAGTTG 1828
                                                   Query:       1 TCTTATATTGAAATCAAC 18
                                                                  ||:|||||| |||||||
Cc_miR-393j   KQ485990.1:701-2755             2.5  Sbjct:      65 AGGATATAAATTTAGTTA 48
                                                   Query:       1 TGAAACTGCCACATGATCT 19
                                                                  |||||||||||||:| |||
Cc_miR-393m   KQ484034.1:19924-20471          1.5  Sbjct:     197 ACTTTGACGGTGTGCAAGA 179
                                                   Query:       1 TGAAATTGAGACTGAGA 17
                                                                  |||||:|:|||||:|||
Cc_miR-393n   CM003606.1:12049408-12058727    1.5  Sbjct:    6244 ACTTTGATTCTGATTCT 6228
                                                   Query:       1 TGAAATTGAGACTGAGA 17
                                                                  |:|| ||||||||||||
Cc_miR-393n   CM003606.1:8961276-8963776      1.5  Sbjct:    1988 ATTTGAACTCTGACTCT 1972
                                                   Query:       1 TGAAATTGAGACTGAGA 17
                                                                  |||||:|||:|||||:|
Cc_miR-393n   KQ483652.1:50842-56011          1.5  Sbjct:    4903 ACTTTGACTTTGACTTT 4887
                                                   Query:       1 TGAAATTGAGACTGAGA 17
                                                                  |||||||||:| |||:|
Cc_miR-393n   CM003604.1:24124885-24126300      2  Sbjct:     487 ACTTTAACTTTTACTTT 471
                                                   Query:       1 TGAAATTGAGACTGAGA 17
                                                                  |||||: :|||||||||
Cc_miR-393n   CM003609.1:2259902-2263480        2  Sbjct:     790 ACTTTGTTTCTGACTCT 774
                                                   Query:       1 TGAAATTGAGACTGAGA 17
                                                                  |||||||||:|||:| |
Cc_miR-393n   CM003613.1:22051457-22052733      2  Sbjct:      84 ACTTTAACTTTGATTAT 68
                                                   Query:       1 TGAAATTGAGACTGAGA 17
                                                                  |||||||| ||||| ||
Cc_miR-393n   KQ484100.1:46125-49497            2  Sbjct:    2376 ACTTTAACCCTGACCCT 2360
```

```
                                                     Query:      1 TGAAATTGAGACTGAGATTGT 21
                                                                   |:|| |||||||||||||| |
Cc_miR-393o   CM003606.1:8961276-8963776       2.5   Sbjct:   1988 ATTTGAACTCTGACTCTAAGA 1968

                                                     Query:      1 TGAATCTCATTAAGGA 16
                                                                   ||||| |||||||:||
Cc_miR-394c   CM003613.1:3565816-3567949       1.5   Sbjct:   1027 ACTTATAGTAATTTCT 1012

                                                     Query:      1 TGAATCTCATTAAGGA 16
                                                                   |||||||||: |||||
Cc_miR-394c   KQ483722.1:45381-50360           1.5   Sbjct:   3919 ACTTAGAGTGCTTCCT 3904

                                                     Query:      1 TGAATCTGGAGGTCCTC 17
                                                                   |||||||| ||||| ||
Cc_miR-394d   CM003605.1:19350860-19353231       2   Sbjct:   1390 ACTTAGACGTCCAGAAG 1374

                                                     Query:      1 TGAATCTGGAGGTCCTC 17
                                                                   ||||||:::|||| |||
Cc_miR-394d   CM003612.1:7239570-7242788       2.5   Sbjct:   1003 ACTTAGGTTTCCATGAG 987

                                                     Query:      1 TGAATCTGGAGGTCCTC 17
                                                                   ||||||::||:||| ||
Cc_miR-394d   KQ483798.1:128238-128876         2.5   Sbjct:    564 ACTTAGGTCTTCAGTAG 548

                                                     Query:      1 TGAATTGCGAGATTTGCA 18
                                                                   ||||||| ||||| ||||
Cc_miR-394f   KQ483840.1:178263-181027           2   Sbjct:     56 ACTTAACACTCTATACGT 39

                                                     Query:      1 TGAATTGCGAGATTTGCA 18
                                                                   ||||||: :|||||||||
Cc_miR-394f   KQ484091.1:4095-6130               2   Sbjct:   1501 ACTTAATCTTCTAAACGT 1484

                                                     Query:      1 TGAATTGCGAGATTTGCA 18
                                                                   ||||||||||:| ||| |
Cc_miR-394f   CM003609.1:410339-419438         2.5   Sbjct:   3601 ACTTAACGCTTTCAACAT 3584

                                                     Query:      1 TGAATTGCGAGATTTGCA 18
                                                                   ||||||||||:|| || |
Cc_miR-394f   KQ483414.1:27433-27928           2.5   Sbjct:    467 ACTTAACGCTTTACACTT 450

                                                     Query:      1 TGAATTGCGAGATTTGCA 18
                                                                   || |:|||:|:|||||||
Cc_miR-394f   KQ483930.1:3858-4647             2.5   Sbjct:    240 ACGTGACGTTTTAAACGT 223
```

```
                                                   Query:          1 TGACTCGTGATATTGG 16
                                                                     ||||||||||:||||
Cc_miR-394i    CM003603.1:11288596-11289715   1.5  Sbjct:        236 ACTGAGCACTGTAACG 221

                                                   Query:          1 TGACTCGTGATATTGG 16
                                                                     ||| ||:||||||||:
Cc_miR-394i    CM003612.1:4498575-4500082       2  Sbjct:         39 ACTAAGTACTATAACT 24

                                                   Query:          1 TGACTCGTGATATTGG 16
                                                                     ||||:|::|||||||:
Cc_miR-394i    KQ483630.1:249064-251799         2  Sbjct:       1620 ACTGGGTGCTATAACT 1605

                                                   Query:          1 TGACTCGTGATATTGG 16
                                                                     || | |||||||||||
Cc_miR-394i    KQ483652.1:191566-195382         2  Sbjct:        299 ACCGTGCACTATAACC 284

                                                   Query:          1 TGAGAGGTGAAGGAAGC 17
                                                                     |||:||||| |||||||
Cc_miR-395a    CM003606.1:7447134-7451410     1.5  Sbjct:       4187 ACTTTCCACCTCCTTCG 4171

                                                   Query:          1 TGAGAGGTGAAGGAAGC 17
                                                                     ||||||||| |:|||||
Cc_miR-395a    KQ483914.1:26581-30139         1.5  Sbjct:       3419 ACTCTCCACGTTCTTCG 3403

                                                   Query:          1 TGAGAGGTGAAGGAAGC 17
                                                                     |||:||||:||| ||||
Cc_miR-395a    CM003605.1:20916010-20917008     2  Sbjct:        806 ACTTTCCATTTCGTTCG 790

                                                   Query:          1 TGAGAGGTGAAGGAAGC 17
                                                                     |||||::|||||||| |
Cc_miR-395a    CM003605.1:28681147-28683483     2  Sbjct:        599 ACTCTTTACTTCCTTGG 583

                                                   Query:          1 TGAGAGGTGAAGGAAGC 17
                                                                     |||:||| |||||||:|
Cc_miR-395a    KQ483441.1:284622-286325         2  Sbjct:       1591 ACTTTCCTCTTCCTTTG 1575

                                                   Query:          1 TGAGAGGTGAAGGAAGC 17
                                                                     |||||||||| |||||
Cc_miR-395a    KQ484000.1:71115-72399           2  Sbjct:        656 ACTCTCCACTCCCTTCA 640

                                                   Query:          1 TGGGTTATTTAAATTTTAACAAAATT 26
                                                                      :||||||||||||||||||||||||
Cc_miR-396ac   CM003605.1:2056377-2061774     1.5  Sbjct:       2158 CTCCAATAAATTTAAAATTGTTTTAA 2133
```

```
                                                        Query:        1 TGTATGGAATGAGAGA 16
                                                                        ||||| ||||||||||
Cc_miR-396ai     KQ484495.1:2952-5894               1   Sbjct:     2236 ACATAACTTACTCTCT 2221

                                                        Query:        1 TGTATGGAATGAGAGA 16
                                                                        |:||||:|||||:|||
Cc_miR-396ai     CM003603.1:8961671-8969816       1.5   Sbjct:     4770 ATATACTTTACTTTCT 4755

                                                        Query:        1 TGTATGGAATGAGAGA 16
                                                                        |:|||:||||||||:|
Cc_miR-396ai     CM003612.1:20398860-20400211     1.5   Sbjct:      801 ATATATCTTACTCTTT 786

                                                        Query:        1 TGTATGGAATGAGAGA 16
                                                                        |:|||:||||||||:|
Cc_miR-396ai     CM003612.1:20398860-20400211     1.5   Sbjct:      801 ATATATCTTACTCTTT 786

                                                        Query:        1 TGTATGGAATGAGAGA 16
                                                                         |||||||||||||:|
Cc_miR-396ai     KQ483498.1:353341-355679         1.5   Sbjct:     1007 CCATACCTTACTCTTT 992

                                                        Query:        1 TGTATGGAATGAGAGA 16
                                                                         |||||||||||||:|
Cc_miR-396ai     KQ483498.1:353341-355679         1.5   Sbjct:     1007 CCATACCTTACTCTTT 992

                                                        Query:        1 TGATGGGAATGTTGTTTGGCT 21
                                                                        |||||:|||||||||| || |
Cc_miR-396d      CM003610.1:9414959-9416240       2.5   Sbjct:      325 ACTACTCTTACAACAACCCAA 305

                                                        Query:        1 TGATGGGAATGTTGTTTGGCT 21
                                                                        |||||:|||||||||| || |
Cc_miR-396d      CM003610.1:9414959-9416240       2.5   Sbjct:      325 ACTACTCTTACAACAACCCAA 305

                                                        Query:        1 TGGATGCAGAGGTTTAT 17
                                                                        ||:|||||||||:||||
Cc_miR-396q      KQ483832.1:118329-119991           1   Sbjct:      535 ACTTACGTCTCCGAATA 519

                                                        Query:        1 TGGATGCAGAGGTTTAT 17
                                                                        ||:|||||||||:||||
Cc_miR-396q      KQ483832.1:118329-119991           1   Sbjct:      535 ACTTACGTCTCCGAATA 519

                                                        Query:        1 TGGATGCAGAGGTTTAT 17
                                                                        ||||||||| :|:||||
Cc_miR-396q      CM003605.1:26949674-26954636       2   Sbjct:      992 ACCTACGTCGTCGAATA 976
```

```
                                                        Query:        1 TGGATGCAGAGGTTTAT 17
                                                                        ||||||||| :|:||||
Cc_miR-396q   CM003605.1:26949674-26954636            2 Sbjct:      992 ACCTACGTCGTCGAATA 976

                                                        Query:        1 TGGATGCAGAGGTTTAT 17
                                                                        ||:|||||:||||| ||
Cc_miR-396q   KQ483438.1:268916-271650                2 Sbjct:     1858 ACTTACGTTTCCAATTA 1842

                                                        Query:        1 TGGATGCAGAGGTTTAT 17
                                                                        ||:|||||:||||| ||
Cc_miR-396q   KQ483438.1:268916-271650                2 Sbjct:     1858 ACTTACGTTTCCAATTA 1842

                                                        Query:        1 TGGATGCAGAGGTTTAT 17
                                                                         ||||||| ||||||||
Cc_miR-396q   KQ483646.1:146077-149392                2 Sbjct:     2209 TCCTACGTATCCAAATA 2193

                                                        Query:        1 TGGATGCAGAGGTTTAT 17
                                                                         ||||||| ||||||||
Cc_miR-396q   KQ483646.1:146077-149392                2 Sbjct:     2209 TCCTACGTATCCAAATA 2193

                                                        Query:        1 TGGATGCAGAGGTTTAT 17
                                                                         :|||:|||||||||||
Cc_miR-396q   KQ483994.1:22970-25497                  2 Sbjct:      173 CTCTATGTCTCCAAATA 157

                                                        Query:        1 TGGATGCAGAGGTTTAT 17
                                                                         :|||:|||||||||||
Cc_miR-396q   KQ483994.1:22970-25497                  2 Sbjct:      173 CTCTATGTCTCCAAATA 157

                                                        Query:        1 TGGCAGAAGAACAGAGAGC 19
                                                                        |::||||||| ||||||||
Cc_miR-396r   KQ483890.1:16708-18081                  2 Sbjct:      385 ATTGTCTTCTCGTCTCTCG 367

                                                        Query:        1 TGGCAGAAGAACAGAGAGC 19
                                                                        |::||||||| ||||||||
Cc_miR-396r   KQ483890.1:16708-18081                  2 Sbjct:      385 ATTGTCTTCTCGTCTCTCG 367

                                                        Query:        1 TGGCTCCCTGTATGCCAT 18
                                                                        ||||||||| |||| ||:
Cc_miR-396u   CM003603.1:4961091-4968047            2.5 Sbjct:     4750 ACCGAGGGAAATACAGTG 4733

                                                        Query:        1 TGGCTCCCTGTATGCCAT 18
                                                                        ||||||||| |||| ||:
Cc_miR-396u   CM003603.1:4961091-4968047            2.5 Sbjct:     4750 ACCGAGGGAAATACAGTG 4733
```

```
                                                          Query:        1 TGGCTCCCTGTATGCCAT 18
                                                                          ||:|||||::|||| |||
Cc_miR-396u    CM003610.1:16337457-16339153          2.5  Sbjct:      766 ACTGAGGGGTATACAGTA 749
                                                          Query:        1 TGGCTGGGGGGAATGA 16
                                                                          ||||||: ||||||||
Cc_miR-396w    KQ483413.1:488486-491362              1.5  Sbjct:      795 ACCGACTACCCTTACT 780
                                                          Query:        1 TGGCTGGGGGGAATGA 16
                                                                          ||:|||| ||||||:|
Cc_miR-396w    KQ483418.1:367701-370414                2  Sbjct:      913 ACTGACCGCCCTTATT 898
                                                          Query:        1 TGGCTGGGGGGAATGA 16
                                                                          |||| ||||||||||
Cc_miR-396w    KQ483489.1:463204-465631                2  Sbjct:      982 ACCGTCCCCCCTTACA 967
                                                          Query:        1 TGGCTGGGGGGAATGA 16
                                                                          ||| |:||||:|||||
Cc_miR-396w    KQ484375.1:12280-13115                  2  Sbjct:      679 ACCTATCCCCTTTACT 664
                                                          Query:        1 TGGCTGGGGGGAATGA 16
                                                                          ||||||: ||||||||
Cc_miR-396x    KQ483413.1:488486-491362              1.5  Sbjct:      795 ACCGACTACCCTTACT 780
                                                          Query:        1 TGGCTGGGGGGAATGA 16
                                                                          ||:|||| ||||||:|
Cc_miR-396x    KQ483418.1:367701-370414                2  Sbjct:      913 ACTGACCGCCCTTATT 898
                                                          Query:        1 TGGCTGGGGGGAATGA 16
                                                                          |||| ||||||||||
Cc_miR-396x    KQ483489.1:463204-465631                2  Sbjct:      982 ACCGTCCCCCCTTACA 967
                                                          Query:        1 TGGCTGGGGGGAATGA 16
                                                                          ||| |:||||:|||||
Cc_miR-396x    KQ484375.1:12280-13115                  2  Sbjct:      679 ACCTATCCCCTTTACT 664
                                                          Query:        1 TGGCTGGGGGGAATGAAGC 19
                                                                          |:|||:|:|||| ||||||
Cc_miR-396y    KQ483427.1:718770-721121              2.5  Sbjct:      260 ATCGATCTCCCTCACTTCG 242
                                                          Query:        1 TGGGAAAAAGTGGA 14
                                                                          | ||||||||||||
Cc_miR-396z    CM003603.1:11288596-11289715            1  Sbjct:      556 AACCTTTTTCACCT 543
```

```
                                                            Query:        1 TGGGAAAAAGTGGA 14
                                                                            |||:||||||||:|
Cc_miR-396z    CM003603.1:14080540-14083865            1   Sbjct:     1200 ACCTTTTTTCACTT 1187
                                                            Query:        1 TGGGAAAAAGTGGA 14
                                                                             |||||||||||||
Cc_miR-396z    CM003604.1:26579219-26580943            1   Sbjct:      415 TCCCTTTTTCACCT 402
                                                            Query:        1 TGGGAAAAAGTGGA 14
                                                                            ||||||||||| ||
Cc_miR-396z    CM003612.1:5550117-5555516              1   Sbjct:     4537 ACCCTTTTTCAACT 4524
                                                            Query:        1 TGGGAAAAAGTGGA 14
                                                                            |||||||||||::|
Cc_miR-396z    KQ483650.1:103002-103376                1   Sbjct:      147 ACCCTTTTTCATTT 134
                                                            Query:        1 TGGGAAAAAGTGGA 14
                                                                            ||| ||||||||||
Cc_miR-396z    KQ483858.1:28722-33061                  1   Sbjct:     1487 ACCATTTTTCACCT 1474
                                                            Query:        1 TTACTAATGTATATAAGTGGA 21
                                                                            :|||| ||:|||||||:||||
Cc_miR-408e    KQ483887.1:113912-135512              2.5   Sbjct:    14018 GATGACTATATATATTTACCT 13998
                                                            Query:        1 TTATATAAGAGAGAATA 17
                                                                            |||||:||||:|:||||
Cc_miR-4995a   AGCT01021301.1:23305-24930            1.5   Sbjct:      915 AATATGTTCTTTTTTAT 899
                                                            Query:        1 TTATATAAGAGAGAATA 17
                                                                            :|||||||||:||||:|
Cc_miR-4995a   CM003605.1:16149916-16150334          1.5   Sbjct:      417 GATATATTCTTTCTTGT 401
                                                            Query:        1 TTATATAAGAGAGAATA 17
                                                                            ||||||||||:|||| |
Cc_miR-4995a   CM003608.1:14907248-14908019          1.5   Sbjct:      547 AATATATTCTTTCTTCT 531
                                                            Query:        1 TTATATAAGAGAGAATA 17
                                                                            | ||||||||||:||||
Cc_miR-4995a   CM003613.1:1577710-1578658            1.5   Sbjct:      512 ATTATATTCTCTTTTAT 496
                                                            Query:        1 TTATATAAGAGAGAATA 17
                                                                            ||||||||||:|| |||
Cc_miR-4995a   CM003613.1:27667222-27671569          1.5   Sbjct:     1216 AATATATTCTTTCCTAT 1200
```

```
                                                      Query:       1 TTATATAAGAGAGAATA 17
                                                                     |||||| |||:|:||||
Cc_miR-4995a   CM003604.1:21520036-21526240       2   Sbjct:    3238 AATATAATCTTTTTTAT 3222

                                                      Query:       1 TTATATAAGAGAGAATA 17
                                                                     |||| |||:|:||||||
Cc_miR-4995a   CM003609.1:2898027-2903029         2   Sbjct:    3162 AATAAATTTTTCTTAT 3146

                                                      Query:       1 TTATATAAGAGAGAATA 17
                                                                     ||||||| ||:|:||||
Cc_miR-4995a   CM003610.1:5679307-5683265         2   Sbjct:    2375 AATATATACTTTTTTAT 2359

                                                      Query:       1 TTATATAAGAGAGAATA 17
                                                                     ||||||||||:|: |||
Cc_miR-4995a   CM003612.1:12363471-12380411       2   Sbjct:   14200 AATATATTCTTTTATAT 14184

                                                      Query:       1 TTATATAAGAGAGAATA 17
                                                                      ||||:||||:||||||
Cc_miR-4995a   CM003612.1:12363471-12380411       2   Sbjct:   10355 TATATGTTCTTTCTTAT 10339

                                                      Query:       1 TTATATAAGAGAGAATA 17
                                                                     |:||||||||:| ||||
Cc_miR-4995a   CM003612.1:21798253-21800184       2   Sbjct:     655 AGTATATTCTTTATTAT 639

                                                      Query:       1 TTATATAAGAGAGAATA 17
                                                                     |||||| |||||||| |
Cc_miR-4995a   KQ484241.1:76435-79891             2   Sbjct:    1148 AATATAATCTCTCTTTT 1132

                                                      Query:       1 TTATATAAGAGAGAATATG 19
                                                                     :|||||||||:||||:||:
Cc_miR-4995b   CM003605.1:16149916-16150334       2   Sbjct:     417 GATATATTCTTTCTTGTAT 399

                                                      Query:       1 TTATATAAGAGAGAATATG 19
                                                                     ||||||||||:| ||| ||
Cc_miR-4995b   CM003606.1:10484971-10486890     2.5   Sbjct:    1368 AATATATTCTTTGTTAAAC 1350

                                                      Query:       1 TTATATAAGAGAGAATATG 19
                                                                     |:||||||||:| |||||:
Cc_miR-4995b   CM003612.1:21798253-21800184     2.5   Sbjct:     655 AGTATATTCTTTATTATAT 637

                                                      Query:       1 TTATATAAGAGAGAATATG 19
                                                                     |||||| |||||||| ||:
Cc_miR-4995b   KQ484241.1:76435-79891           2.5   Sbjct:    1148 AATATAATCTCTCTTTTAT 1130
```

```
                                                         Query:      1 TTATATAAGAGAGAATATG 19
                                                                       :|| |||||||||||| ||
Cc_miR-4995b   KQ484298.1:72775-75219              2.5   Sbjct:    211 GATTTATTCTCTCTTACAC 193

                                                         Query:      1 TTATATAAGAGAGAATATGA 20
                                                                       ||||||||||:| ||| |||
Cc_miR-530a    CM003606.1:10484971-10486890        2.5   Sbjct:   1368 AATATATTCTTTGTTAAACT 1349

                                                         Query:      1 TTATATAAGAGAGAATATGA 20
                                                                       |||||| |||||||| ||:|
Cc_miR-530a    KQ484241.1:76435-79891              2.5   Sbjct:   1148 AATATAATCTCTCTTTTATT 1129

                                                         Query:      1 TTGACAGAAGAGAGTGAGCAC 21
                                                                       |||||||||||||| ||||||
Cc_miR-8005a   CM003604.1:15225003-15228624          1   Sbjct:   2715 AACTGTCTTCTCTCTCTCGTG 2695

                                                         Query:      1 TTGACAGAAGAGAGTGAGCAC 21
                                                                       ||||||||||| || ||||||
Cc_miR-8005a   CM003608.1:4650408-4651927            2   Sbjct:   1283 AACTGTCTTCTATCTCTCGTG 1263

                                                         Query:      1 TTGACAGAAGAGAGTGAGCAC 21
                                                                        ||||||| ||||||||||||
Cc_miR-8005a   CM003612.1:3998296-3999928            2   Sbjct:   1433 CACTGTCTGCTCTCACTCGTG 1413

                                                         Query:      1 TTGACAGAAGAGAGTGAGCAC 21
                                                                       ||||||||||| || ||||||
Cc_miR-8005a   KQ483957.1:13066-15078                2   Sbjct:    276 AACTGTCTTCTATCTCTCGTG 256

                                                         Query:      1 TTGACAGAAGAGAGTGAGCAC 21
                                                                       ||||||||||| || :|||||
Cc_miR-8005a   CM003609.1:18479681-18480594        2.5   Sbjct:    753 AACTGTCTTCTATCTTTCGTG 733

                                                         Query:      1 TTGGTGAGGTTCAATC 16
                                                                       :|||||| ||||||||
Cc_miR-9730c   CM003606.1:2283227-2286128          1.5   Sbjct:     47 GACCACTACAAGTTAG 32

                                                         Query:      1 TTGGTGAGGTTCAATC 16
                                                                       ::|||||||||||||
Cc_miR-9730c   CM003608.1:21273393-21278583          2   Sbjct:   5053 GGCCACTCCAAGTTAA 5038

                                                         Query:      1 TTGGTGAGGTTCAATC 16
                                                                       :||:||||:|||||:|
Cc_miR-9730c   CM003609.1:14350001-14360764          2   Sbjct:   6992 GACTACTCTAAGTTGG 6977
```

```
                                                          Query:       1 TTGGTGAGGTTCAATC 16
                                                                         |||:|:|| |||||||
Cc_miR-9730c    KQ483469.1:394906-406708              2   Sbjct:    2468 AACTATTCAAAGTTAG 2453

                                                          Query:       1 TTGGTGAGGTTCAATC 16
                                                                         ||||||||| |||||
Cc_miR-9730c    KQ483504.1:308351-313777              2   Sbjct:    2165 AACCACTCCTAGTTAT 2150

                                                          Query:       1 TTGGTGAGGTTCAATC 16
                                                                         :|:||||| |||||||
Cc_miR-9730c    KQ483562.1:264986-268393              2   Sbjct:     322 GATCACTCAAAGTTAG 307

                                                          Query:       1 TTGGTGAGGTTCAATC 16
                                                                          ||||| |||||||||
Cc_miR-9730c    KQ483808.1:14390-16208                2   Sbjct:     519 TACCACACCAAGTTAG 504

                                                          Query:       1 TTGGTGAGGTTCAATC 16
                                                                          |:|:|||||||||||
Cc_miR-9730c    KQ484138.1:84579-85395                2   Sbjct:     195 CATCGCTCCAAGTTAG 180

                                                          Query:       1 TTTAAGAGAGATTTATCA 18
                                                                         |:|||||||||:||| ||
Cc_miR-9749a    KQ483441.1:284622-286325              2   Sbjct:     851 AGATTCTCTCTGAATCGT 834

                                                          Query:       1 TTTAAGAGAGATTTATCA 18
                                                                         ||||||||||||| |||
Cc_miR-9749a    KQ484271.1:91081-92342                2   Sbjct:      68 AAATTCTCTCTAATTAGA 51

                                                          Query:       1 TTTCTTGACATTGCTTGTTGCTT 23
                                                                         ||||||||||| |||||:| |||
Cc_miR-9756b    KQ484825.1:1639-2869                2.5   Sbjct:     874 AAAGAACTGTATCGAACGAAGAA 852

                                                          Query:       1 TTTTCTCTGAGGAATTA 17
                                                                         ||||||||||::|||:|
Cc_miR-9756e    AGCT01017205.1:911-2412             1.5   Sbjct:    1413 AAAAGAGACTTTTTAGT 1397

                                                          Query:       1 TTTTCTCTGAGGAATTA 17
                                                                         |||||||||| :|||||
Cc_miR-9756e    KQ483735.1:15946-19585              1.5   Sbjct:    2581 AAAAGAGACTATTTAAT 2565

                                                          Query:       1 TTTTCTCTGAGGAATTA 17
                                                                         ||:||||| ||:|||||
Cc_miR-9756e    AGCT01020320.1:4915-15191             2   Sbjct:    7018 AAGAGAGAGTCTTTAAT 7002
```

```
                                                                   Query:         1 TTTTCTCTGAGGAATTA 17
                                                                                    |||  ||||||||||||
Cc_miR-9756e    CM003604.1:19500978-19504286                    2  Sbjct:      1618 AAACCAGACTCCTTAAT 1602
                                                                   Query:         1 TTTTCTCTGAGGAATTA 17
                                                                                    ||:|||||:|| |||||
Cc_miR-9756e    CM003604.1:21520036-21526240                    2  Sbjct:      1941 AAGAGAGATTCATTAAT 1925
                                                                   Query:         1 TTTTCTCTGAGGAATTA 17
                                                                                    ||||||||||:| |:||
Cc_miR-9756e    CM003613.1:1835147-1838048                      2  Sbjct:      2452 AAAAGAGACTTCGTGAT 2436
                                                                   Query:         1 TTTTCTCTGAGGAATTA 17
                                                                                    |||||: ||||:|||||
Cc_miR-9756e    KQ483415.1:412027-418648                        2  Sbjct:      1094 AAAAGGTACTCTTTAAT 1078
                                                                   Query:         1 TTTTCTCTGAGGAATTA 17
                                                                                    |||||: ||||:|||||
Cc_miR-9756e    KQ483415.1:412027-418648                        2  Sbjct:       954 AAAAGGTACTCTTTAAT 938
                                                                   Query:         1 TTTTCTCTGAGGAATTA 17
                                                                                    :|:||||||||||| ||
Cc_miR-9756e    KQ483493.1:37655-38441                          2  Sbjct:       453 GAGAGAGACTCCTTTAT 437
                                                                   Query:         1 TTTTCTCTGAGGAATTA 17
                                                                                     ||||||||||||| ||
Cc_miR-9756e    KQ483496.1:140155-142806                        2  Sbjct:       446 CAAAGAGACTCCTTCAT 430
                                                                   Query:         1 TTTTCTCTGAGGAATTA 17
                                                                                     |||||||:||||||:|
Cc_miR-9756e    KQ483744.1:113754-114374                        2  Sbjct:       341 CAAAGAGATTCCTTAGT 325
                                                                   Query:         1 TTTTCTCTGAGGAATTA 17
                                                                                    |||||||| |:|||:||
Cc_miR-9756e    KQ483909.1:114218-119419                        2  Sbjct:      1481 AAAAGAGAATTCTTGAT 1465
                                                                   Query:         1 TTTTCTCTGAGGAATTA 17
                                                                                     |||||||:||||||:|
Cc_miR-9756e    KQ484661.1:28487-29994                          2  Sbjct:      1444 CAAAGAGATTCCTTAGT 1428
                                                                   Query:         1 TTTTCTCTGAGGAATTA 17
                                                                                    |||| |||:|:||||||
Cc_miR-9756e    KQ484830.1:10546-11548                          2  Sbjct:       777 AAAAAAGATTTCTTAAT 761
```

Supplementary S12. Primer name and sequences used in this study.

NAME	SEQUENCE(5'-3')
Cc_lncRNA-510-F	TCCGCGTACATGCGATTT
Cc_lncRNA -510-R	ACAGTGGGTGAAAGTGGAAG
Cc_lncTAR-510-F	CCTGGATGGAGAGATCTTTGAAC
Cc_lncTAR-510-R	TGTGATGCTGGCGAATAACT
Cc_lncRNA -604-F	GACGGAGTATGCTGGAGATAAG
Cc_lncRNA -604-R	TGCATGTGGCTCGTCTTT
Cc_lncTAR-604-F	GGAGGAGACAAGTTGGTCATAAA
Cc_lncTAR-604-R	GACGACGCTCCACATGAAT
Cc_lncRNA -765-F	ACACTGCTTGACATGATTTGTG
Cc_lncRNA -765-R	CTCCGAATCTTCCTTCGTCTTT
Cc_lncTAR-765-F	AGTATACAAGGGAGGCCTAACA
Cc_lncTAR-765-R	GAGGAGTGCCCTCTAGGAAATA
Cc_lncRNA-810-F	GGAGATGGTTCGGAGGTTAATG
Cc_lncRNA-810-R	TCCTCCGCTAAGGAAGTGT
Cc_lncTAR-810-F	AGAGGAAGTGGGAAGGATTTG
Cc_lncTAR-810-R	TTCCACGTCAGATTATCCTCTTC
Cc_lncRNA-902-F	ACTCCGCTGCTTCTATGAAC
Cc_lncRNA-902-R	GAGAGGTCCAACCCAGTAAAC
Cc_lncTAR-902-F	ACTCCGCTGCTTCTATGAAC
Cc_lncTAR-902-R	GAGAGGTCCAACCCAGTAAAC
Cc_lncRNA-1331-F	AGGTGGAGGAGGAAGTGTAA
Cc_lncRNA-1331-R	TGCTAGACTACCAACACTTTCAG
Cc_lncTAR-1331-F	AGGTGGAGGAGGAATTGTAGA
Cc_lncTAR-1331-R	AATGCTACCACCTCCTTTGG
Cc_lncRNA-1516-F	GGAGGTTGGTCAAGACATACAG
Cc_lncRNA-1516-R	ACTCGCATCCCTTGCATAAG

Cc_lncTAR-1516-F	ACAACTGCAACAACAGCAATAG
Cc_lncTAR-1516-R	TGAGAAGTGCAACCCAAGAG
Cc_lncRNA-1660-F	CCCGTGCTTCTACAACAATCT
Cc_lncRNA-1660-R	CACAAGAGGTCATCATCCTTCTC
Cc_lncTAR-1660-F	CCCGTGCTTCTACAACAATCT
Cc_lncTAR-1660-R	CACAAGAGGTCATCATCCTTCTC
Cc_lncRNA-1896-F	GGACACGAATGTGACCGTATT
Cc_lncRNA-1896-R	CAGAGAGTCAGAGCCACAAAG
Cc_lncTAR-1896-F	AGGGTAAACATGGTCCTTTCTC
Cc_lncTAR-1896-R	GACGCTCACTTGAGCTCTTAT
Cc_lncRNA2150-F	AAGAGTGGAAGGCGTTGTG
Cc_lncRNA2150-R	TGTACCTCCCTTCGGTTAAGA
Cc_lncTAR-2150-F	CCACTGGGTATGCTGAAACTAA
Cc_lncTAR-2150-R	CAACACGTAGACGTCATCAGTAA
Cc_lncRNA-2482-F	TGTTGTCTACCGGTGCTTATATC
Cc_lncRNA-2482-R	CCGATAAGCAGCATGGAACTA
Cc_lncTAR-2482-F	AGCACCAATGCTAGCTCTAAG
Cc_lncTAR-2482-R	CGAAAGCAAGCTGCTGAATG
Cc-miR-160h	AGGTTAAGGTGGAGAT
XM_020377020-F	TCTTAAGCAAACCGGAGAAGAG
XM_020377020-R	TGTGATCCAGACCCTTCTATCT
Cc-miR160h-tarLNC-F	GGGAAGTGAAGTTTGAGGTTTG
Cc-miR160h-tarLNC-R	CGTTCCCTCTCTTTCTCCTTT
Cc-miR-164h	ATCATCAAGATTCTCA
XM_020382865-F	TTGATTGGTGAGGAAGATCAGG
XM_020382865-R	CTGAGAGAATGGCAGGAATGT
Cc-miR164h-tarLNC-F	CTCTAGGAGCTTGTTTGAGACTAT
Cc-miR164h-tarLNC-R	GTGAGACAACCTTATGGCTTTG
Cc-miR-164i	ATCCAAAGGGATCGCATTGATCCTA

XM_020371081-F	TGACGGACGTAGGGAAGTAT
XM_020371081-R	ATCTCCACATTCAGCCTTGG
Cc-miR164i-tarLNC-F	CCTCCAGTTGCAGTGGAATA
Cc-miR164i-tarLNC-R	GAAAGAGAACGAGAGAGGAAGG
Cc-miR-319ai	TATGGAATGAGAGAGACAA
XM_020376546-F	TCGTTAGTGGTTGGTGTTCTC
XM_020376546-R	TAGCGAGAGAGAGCCAGAAT
Cc-miR319ai-tarLNC-F	GAAGAACAACCCAATCCTCAAATC
Cc-miR319ai-tarLNC-R	GAATCCTCATACCAAGTGTCTACC
Cc-miR156as	AGCATGATCCCAAA
XM_020364576-F	TGCGATTTGGCACAGGATAG
XM_020364576-R	GAACTGGCTAACACACTGTAGG
Cc-miR156as-tarLNC-F	CTCACGGTCTTGATCTGTTCAT
Cc-miR156as-tarLNC-R	GCTGTGGATGTTGTCTCCTAC
Cc-miR-166z	CCACATTTTTCTTAAAC
XM_020347729-F	GACAGACCTTGCTCGTTCTT
XM_020347729-R	GGCTAGCGGTGGTTATTTCT
Cc-miR166z-tarLNC-F	GAACGAATGTGGTGTGTTCTTC
Cc-miR166z-tarLNC-R	GGCATGGAGTGGATGAGAAA
Cc-miR-9756e	TTTTCTCTGAGGAATTA
XM_020376117-F	GTGATGGCTTGGCAGAAAGA
XM_020376117-R	GTCGAGTCAACGATCCAATCAG
Cc-miR9756e-tarLNC-F	GTTGGAGGGAGAGAAGGAATTG
Cc-miR9756e-tarLNC-R	CCCATTTCCTGAATCCCTAGTT
Cc-miR-156aq	AGCATCATCAAGATTC
XM_020384165-F	TGCCCGAGACAAAGAACATC
XM_020384165-R	ATGAACCACCGGCTACAATC
Cc-miR156aq-tarLNC-F	CCTGAGAGGCAACAAGTAAGT
Cc-miR156aq-tarLNC-R	CGGGATAGGAGCTTGCTAAAT

Cc-miR-166f	ATTTTCTCTCTACGTT
XM_020363260-F	AAGTCTCACATCAGTGGATTAACA
XM_020363260-R	ATTAGACTACGCGAGCTTTGG
Cc-miR166f-tarLNC-F	GCAGATTCGGACTTGCCATA
Cc-miR166f-tarLNC-R	CTGCTTCTCTCTCCTCTGTTTC
